# First Report of Pathogenic Bacterium *Kalamiella piersonii* Isolated from Urine of a Kidney Stone Patient: Draft Genome and Evidence for Role in Struvite Crystallization

**DOI:** 10.3390/pathogens9090711

**Published:** 2020-08-29

**Authors:** Punchappady Devasya Rekha, Asif Hameed, Muhammed A. P. Manzoor, Mangesh V. Suryavanshi, Sudeep D. Ghate, A. B. Arun, Sneha S. Rao, Athmika, Sukesh Kumar Bajire, M. Mujeeburahiman, C.-C. Young

**Affiliations:** 1 Yenepoya Research Centre, Yenepoya Deemed to be University, Mangalore 575018, India; asifbiology@gmail.com (A.H.); manzoorapkdy@gmail.com (M.A.P.M.); mangeshnccs@gmail.com (M.V.S.); sudeep1129@gmail.com (S.D.G.); bhagwatharun@hotmail.com (A.B.A.); sneharao16694@gmail.com (S.S.R.); athmikashetty23@gmail.com (A.); sukeshindra@gmail.com (S.K.B.); 2 Department of Soil and Environmental Sciences, College of Agriculture and Natural Resources, National Chung Hsing University, Taichung 40227, Taiwan; ccyoung@mail.nchu.edu.tw; 3 Department of Urology, Yenepoya Medical College and Hospital, Yenepoya Deemed to be University, Mangalore 575018, India; mujeeburahiman@gmail.com

**Keywords:** uropathogen, amino acid metabolism, asparaginase, urea carboxylase, struvite, media alkalization, virulence, synthetic urine, motility and biofilm, lithogenesis

## Abstract

Uropathogenic bacteria are widely distributed in the environment and urinary tract infection is implicated in kidney stone disease. Here, we report on a urease negative bacterium *Kalamiella piersonii* (strain YU22) isolated from the urine of a struvite stone (MgNH_4_PO_4_·6H_2_O) patient. The closest species, *K. piersonii* IIIF1SW-P2^T^ was reported from International Space Station samples. However, there are no earlier reports on its human association. Using whole genome and experimental analysis, its involvement in urinary tract colonization and struvite crystallization was explored. The strain YU22 showed many virulence factors that are needed for host cell invasion and colonization including cell adhesion factors, swimming and swarming motilities, biofilm and siderophore among others. In vitro infection studies in HEK-293T cells demonstrated the host cell attachment and killing. It was able to utilize amino acids as sole carbon source and showed growth in synthetic and healthy urine establishing metabolic adaptation to urinary tract. Increased pH and availability of ammonium ions from amino acid breakdown promoted struvite crystallization. The results from this study support the involvement of urease negative uropathogen in the struvite lithogenesis. Further studies on other isolates of *K. peirsonii* are warranted to assess its health risks.

## 1. Introduction

Kidney stone disease is one of the most common urological problems worldwide, and stone formation process involves crystal nucleation, aggregation and/or secondary nucleation, fixation within the kidney or renal collecting system and growth [1,2]. Super-saturation of urine, urinary pH, ionic strength, specific gravity, concentration of solutes of urine, and infection are the major factors contributing to stone formation. Among the different types of kidney stones, magnesium ammonium phosphate hexahydrate (MgNH_4_PO_4_·6H_2_O) also known as struvite (7–15% of all stone types) is mainly associated with UTI by urease positive bacteria such as, *Proteus*, *Klebsiella*, *Staphylococcus*, *Providencia* [3]. The stone can grow rapidly occupying the renal calyces and pelvis damaging significantly the epithelium of the internal renal walls [4]. Data on the bacteriology of struvite stone cultures and urine cultures agree largely on the presence of infection agents [5,6]. Patients with kidney stone disease are more likely to have urinary tract infection (UTI) due to the invasion of uropathogens and their colonization in bladder or ascending infection to kidney.

Bacterial colonization in the urinary tract requires well established virulence mechanisms to attach, invade and survive in the urinary tract environment. Invasion and survival require several adaptations involving epithelial attachment, host cell invasion and intracellular proliferation ability, flexible metabolic pathways along with ability to disseminate and re-infect neighbouring epithelial cells [7,8,9]. The genomic data and experimental evidences indicate that the successful uropathogens have well developed cell adhesive structures such as pili and adhesins, surface proteins for helping in facilitating attachment and engulfment into host cell and biofilm forming ability among other virulence factors [10,11,12].

During our investigations on the urine microbiome of kidney stone patients, we isolated a unique bacterial strain from the urine of a struvite stone patient designated as YU22 (=MCC3118). It was identified at the time of isolation as *Pantoea* sp. based on the available sequences in the NCBI database. Subsequently, it was identified as *Kalamiella piersonii* with 100% 16S rRNA gene sequence similarity with type strain IIIF1SW-P2^T^ that was isolated from the International Space Station (ISS) [13]. Computational analysis of IIIF1SW-P2^T^ provided no evidence to suggest its human colonization, and there are no reports elsewhere on its human association. However, its closely related genera have been established as human pathogens with reported cases of infections and they include; *Erwinia* [14] and *Pantoea* [15]. The other closely related genus *Mixta* also has species having association with humans, though the pathogenesis is still not well established [16]. Some studies also suggest that many clinical isolates of *Pantoea* are not identified properly; for example, Soutar et al. [17] using MALDI-TOF based identification reclassified a clinical isolate of *Pantoea* as *Mixta calida*. Based on this information, we sequenced the genome of the strain YU22 and performed culture-dependant analysis to understand its clinical significance. The genome was mined for virulence genes and metabolic pathways that can provide thorough insights into its ability to invade the host and urinary tract colonization. The data validated using laboratory experiments provide preliminary evidence for its involvement in human colonization and is suggestive of role in initial crystallization of struvite.

## 2. Results

### 2.1. The Patient Demographics and Bacterial Characteristics

The bacterium YU22 was isolated from the urine of a male patient with symptomatic kidney stone disease. The patient reported symptoms such as severe pain and difficulty in passing urine. Clinical investigations suggested the kidney stone. The urine pH was 7.0, blood urea and serum creatinine levels were 20 mg/dL (reference range: 7.0–22 mg/dL) and 1.4 mg/dL (reference range 0.7–1.2 mg/dL), respectively, and patient did not have an active UTI. Detailed blood and urine analyses are given in supplementary table (Table S1). The mineral composition of the surgically removed kidney stone was identified based on the FT-IR as pure struvite. The urine was culture positive, and the isolate YU22 was collected. It showed rapid growth in nutrient agar (NA), and colonies were off-white in colour and semi-translucent (Figure S1a). The bacterium was Gram negative and rod shaped with a size ~1 µ (Figure S1b). The pH tolerance studies showed strain YU22 was able to grow in the pH range of 5–10. The carbon source utilization and the enzyme details from the Biolog and API systems are given as supplementary document (S1).

### 2.2. Taxonomic Identity and Draft Genome of *Kalamiella piersonii* YU22

**Figure 1:**
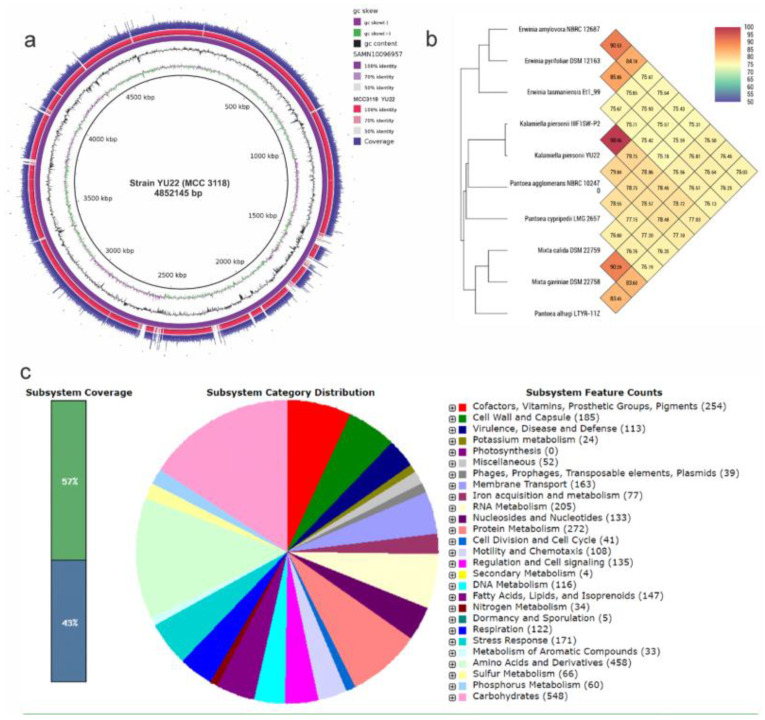
Draft genome sequence analysis of the clinical isolate *Kalamiella piersonii* YU22: (**a**) circular diagram showing draft genome map, (**b**) OrthoANI heat map showing the genomic similarity of YU22 with *K. piersonii* IIIF1SW-P2^T^, isolated from International Space Station and other related type strains and (**c**) subsystem coverage of the genome of YU22 as annotated by Rapid Annotations using Subsystems Technology (RAST). (YU22 = MCC3118).

The 16S rRNA gene sequence analysis at EzBiocloud database indicated that YU22 showed 100% similarity to *Kalamiella piersonii* IIIF1SW-P2^T^ (Table S2). The strain YU22 shared >97.0% 16S rRNA gene sequence similarity with type strains of *Pantoea* (*n* = 13, 97.1–97.9%), *Erwinia* (*n* = 2, 97.1–97.3), *Mixta* (*n* = 2, 97.1–97.2%), *Flavobacterium* (*n* = 1, 97.6%) and *Klebsiella* (*n* = 1, 97.2%). Thus, whole genome sequencing was carried out to identify the exact taxonomic status of this new strain. The draft genome size was approximately 4.79 Mb; the genome assembly consisted of 55 contigs, 4699 coding sequences, 83 genes encoding RNA and G + C content of 57.3% (Figure 1a). Based on the genome relatedness index, measured as the OrthoANI, YU22 shared the highest genome similarity (99%) to the type strain *K. piersonii* IIIF1SW-P2^T^ and showed OrthoANI scores of 75.6–79.8% to other related strains (Figure 1b). The common subsystem structure of the genome consisted of carbohydrates (548), protein metabolisms (272), virulence, disease and defence (113), stress response (171) and other genes (Figure 1c). The detailed list of functional categories obtained from the COG database is given in Supplementary (Table S3).

### 2.3. Bacterial Adherence Factors, Motility and Biofilm Formation Involved in Virulence

**Figure 2:**
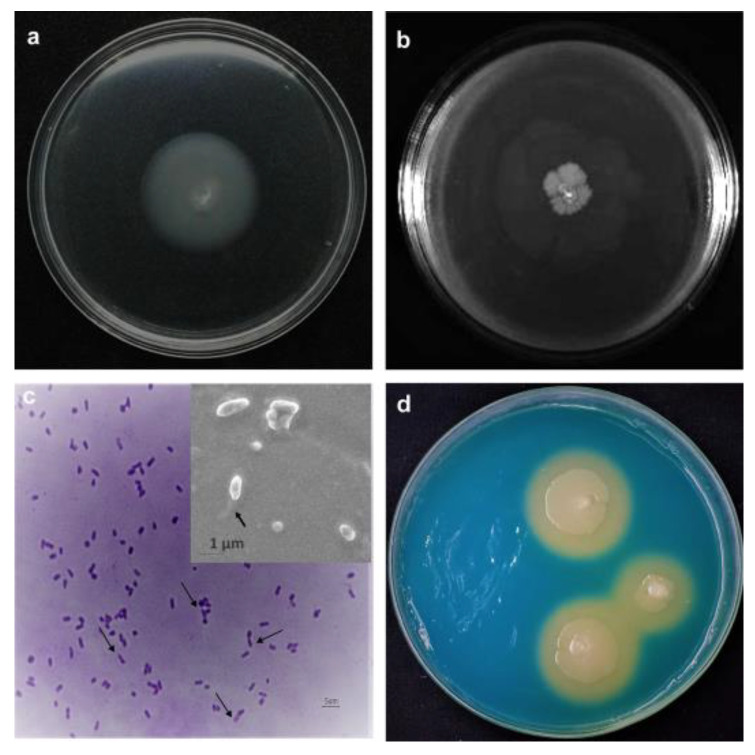
Virulence factors of *Kalamiella piersonii* YU22: (**a**) swimming motility on 0.3% agar observed after 24 h of incubation, (**b**) swarming motility in 0.5% agar observed at 96 h, (**c**) flagella demonstrated by flagellar staining and inlet SEM image showing the flagella (black arrows point the flagella), (**d**) siderophore production on chrome azural S (CAS) agar plate incubated for 96 h. Change in colour around the colonies to orange indicate the siderophore production.

The YU22 genome data showed many virulence factors including genes coding for adherence factors, chemotaxis and motility (Table S4). The adherence factors (adhesins) are important to form and maintain physical interactions with host cells and tissues. Among these; eight *che* genes, two *mot* genes (*motA* and *motB*), 14 *flg* genes, four *flh* genes, and 21 *fli* genes encoding the components of the basal body and the flagellar export apparatus are found. Genes of Type IV pili (*pilA*, *pilB*, *pilC* and *pilD*) and fimbriae-like adhesin (*SfmA*) indicate the high motility of the bacterium that is required for host cell attachment and invasion. The genomic data was validated with different motility experiments in the laboratory. The bacterium showed very high motility in the liquid media and in the motility test agar. In the swimming agar plate, the colony expanded rapidly reaching 30 mm diameter within 24 h (Figure 2a). The swarming motility was slower compared to swimming and reached significant colony diameter on prolonged incubation of 96 h (Figure 2b). The bacteria formed biofilm on the polystyrene surface under static incubation condition with a biofilm intensity represented by OD_590_ of 0.17 ± 0.01 at 24 h and 0.24 ± 0.01 at 48 h. Microscopic observations of the stained cells showed flagellar organization with the presence of lophotrichous flagella located at one end of bacteria and also the same was observed in the scanning electron microscopy (Figure 2c).

#### 2.3.1. Siderophore Production and Iron Uptake

Siderophore is a well-known virulence factor needed for iron acquisition and helps in compete with the host cells for iron. The genome data showed the genes for iron uptake and transport system such as *fepB*, *fepD* and *fepG* and was validated using the chrome azural S (CAS) agar assay. The CAS agar plates showed change in colour from blue to orange due to the siderophore production (Figure 2d).

#### 2.3.2. Serum Killing, Host Cell Attachment and Cell Killing

**Figure 3:**
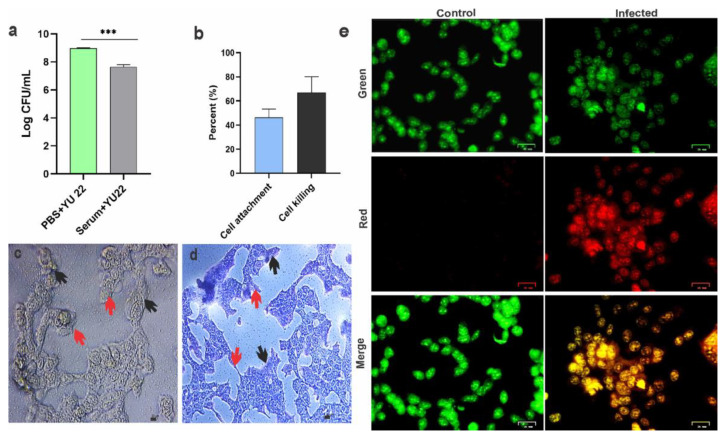
Serum sensitivity and in vitro cell infectivity of *Kalamiella piersonii* YU22: (**a**) serum killing effect of YU22 compared to control (mean ± SD, *n* = 3, *** *p* < 0.001). Bacteria were incubated with healthy human serum or PBS (Control) for 30 min and the CFU were calculated by serial dilution method. (**b**) Percentage host cell attachment and killing. HEK 293T cells were infected with YU22 (MOI of 10) and the CFUs were calculated for estimating the cell attachment. For comparison bacteria without host cells were incubated. (**c**,**d**) Micrographs showing bacterial attachment to HEK293T cells in bright field and with Giemsa stain, respectively. Black arrows indicate bacteria and Red arrows showing damaged cells. (**e**) Live/dead staining of HEK 293T cells infected with YU22 in comparison to uninfected control. Images show live (green) and apoptotic (red) cells.

The serum kill assay was performed to establish the sensitivity of YU22 to healthy serum. We observed a significant reduction by 20% in cell viability compared to control group incubated with PBS (Figure 3a). The ability of the bacteria to attach to the human cells was tested in vitro using HEK293T cells. The bacteria formed a strong attachment with 46.5% of bacteria showing colonization/attachment on the HEK293T cells and caused 67% cell killing calculated based on the trypan blue dye exclusion method (Figure 3b). The cell attachment visualized by microscopy and Giemsa staining technique showed the bacteria on the surface and intercellular spaces of the HEK293T cells (Figure 3c,d). The morphological changes in the cells caused by the bacteria included shrinkage in the size possibly with some effect on cytoskeleton. The fluorescent live dead staining showed apoptotic cells in the YU22 infected HEK293T cells (Figure 3e).

### 2.4. Urea Metabolismin *K. piersonii* YU22

The genome of *K. piersonii* YU22 was screened for the genes involved in the metabolism of urea, which is a predominant excretory product found in human urine. The strain YU22 lacked all three subunits (α, β and γ) of urease, which is similar to strain IIIF1SW-P2^T^. The genome data mining showed the alternate urea breakdown enzyme system consisting of the urea carboxylase (UC; EC 6.3.4.6) and allophanate hydrolase (AH; EC 3.5.1.54) in both the strains. The phylogenetic analysis based on the full-length amino acid sequence showed close affiliation of UC to fungal urea amidolyases (UALs) whereas AH formed a distinct and separate clade displaying heterogeneity in the distribution of UC and AH among the members of family *Erwiniaceae* (Figure S2). The phylogenetic data further revealed the phyletic clustering of UAL of *Pantoea ananatis* (sole bacterium reported to bear UAL [18]) with bacterial AHs. The genes in the biotin-dependent UC and AH pathways for the urea breakdown are given as supplementary table (Table S5).

**Figure 4:**
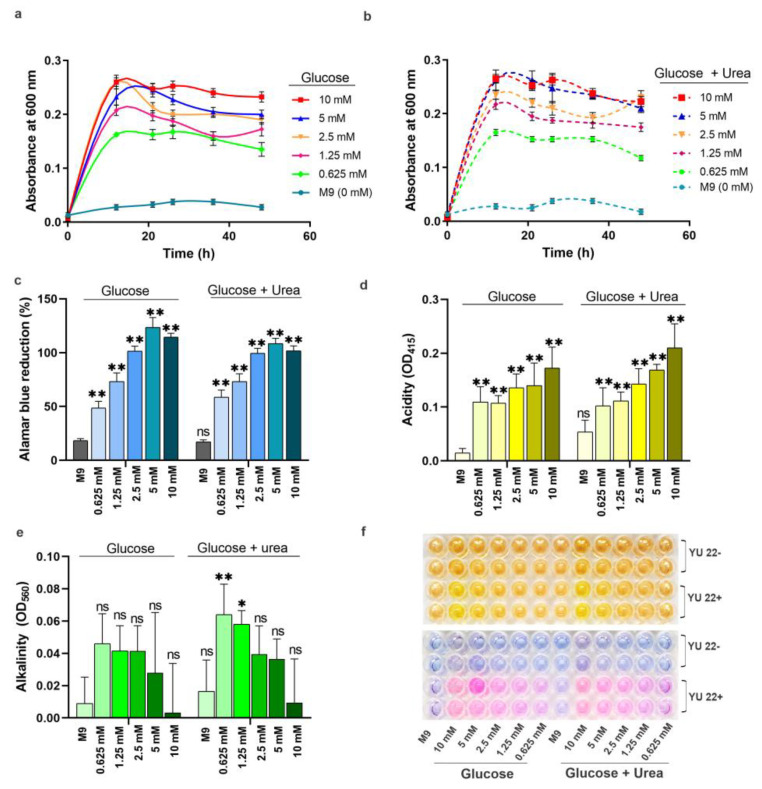
Impact of urea and glucose on the growth kinetics, media pH and metabolism of *Kalamiella piersonii* YU22: (**a**) growth curves of the bacteria grown at varying concentration of glucose and (**b**) same coloured dotted lines represent the respective glucose treatments supplemented with 10 mM urea. (**c**) Colorimetric determination of cell metabolism, (**d**) media acidity, (**e**) media alkalinity and (**f**) sections of microplates showing colour formation after the addition of phenol red (upper panel) and alamar blue reagents (lower panel). Media acidity and alkalinity were measured after addition of phenol red as OD_415_ and OD_550_, respectively. YU22-, cells absent; YU22+, cells present. Data points are mean ± SD (*n* = 4). * *p* < 0.05, ** *p* < 0.01, ns: not significant compared to M9 media.

To see whether the urea containing media supports bacterial growth, YU22 was grown in M9 media with different concentrations of urea. The growth in M9 minimal media was supplemented with varying concentrations of glucose and 10 mM urea and as comparator with only glucose is illustrated in Figure 4a,b. The metabolic activities tested based on the alamar blue reduction assay showed higher activity at higher levels of glucose and no difference in presence of urea except for the lowest concentration of glucose was observed (Figure 4c). The acidity of the media increased with increasing concentration of glucose despite the presence or absence of urea (10 mM) (Figure 4d). The alkalinity increased significantly with lower concentration of glucose in the presence of urea (Figure 4e,f).

### 2.5. Amino Acid Catabolism, Ammonia Formation and Media pH

**Figure 5:**
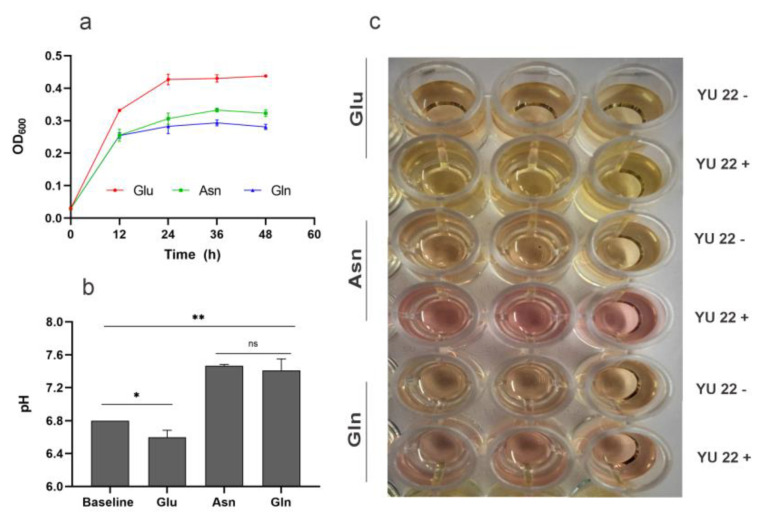
Growth and pH evolution observed in the cultures of *Kalamiella piersonii* YU22 in amino acid supplemented media: (**a**) growth curve, (**b**) media pH at 48 h measured using a digital pH electrode. The basal pH of M9 media was set at 6.8. (**c**) Sections of the 96-well plates showing colour formation after the addition of phenol red indictor. α-D-glucose (Glu), L-asparagine (Asn) and glutamine (Gln) 10 mM each in M9 basal were used. Data points are mean ± SD (*n* = 3). * *p* < 0.05, ** *p* < 0.01, ns: not significant.

Uropathogens often have the ability to utilize the amino acids and hence, the genomes were mined for the presence of enzyme for amino acid degradation. We found genes coding for asparaginase (EC 3.5.1.1) and glutaminase (EC 3.5.1.2) and a multispecies cytosolic Asn/Gln permease in both the strains of *K. piersonii*. Based on this, growth experiments were carried out for YU22 using asparagine and glutamine(10 mM) as sole carbon sources, and the growth trend was compared with that of glucose in M9 minimal media (Figure 5a). The bacterial growth followed a sigmoidal pattern in amino acid containing media, whereas bacterial growth was more rapid in the presence of glucose. The media pH measured after 48 h incubation was significantly higher (*p* < 0.01) in the presence of 10 Mm asparagine and glutamine, compared to that with glucose (Figure 5b,c). Further, amino acid supplementation increased the media alkalization during the growth of YU22.

**Figure 6:**
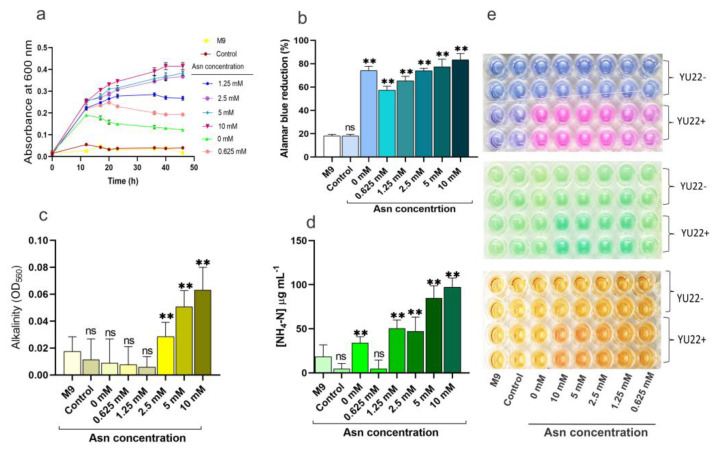
Growth kinetics, media alkalinity, extracellular ammonium discharge and metabolism in *Kalamiella piersonii* YU22 grown with varying doses of L-asparagine (Asn) in M9 media containing 10 mM urea and 0.625 mM glucose: (**a**) differential growth curves, (**b**) metabolism, (**c**) alkalinity determined by colorimetric method and (**d**) NH_4_-N concentration in the culture incubated for 24 h. (**e**) Sections of microplate after the addition of alamar blue dye, NH_4_-N reagent and phenol red (top to bottom). YU22- without cells; YU22+, with cells; M9, without carbon supplement, control: M9 media with 10 mM urea; Other data points (0 mM–10 mM) represent M9 media supplemented with 10 mM urea, 0.625 mM glucose and varying concentrations of asparagine; data points are mean ± SD (*n* = 4); ** *p* < 0.01, ns: not significant compared to control.

To check whether the media pH was due to the ammonium ions released from the breakdown of amino acids, ammonia production was monitored with different concentrations of a selected amino acid (L-asparagine). The growth was highly influenced by the amino acid levels and higher growth rates (OD_600_) were observed with increasing concentration of asparagine in the media (Figure 6a). The metabolic activities were similarly higher with higher amino acid concentration in the media (Figure 6b). Increased alkalinity and ammonia production were observed with increasing concentrations of asparagine (Figure 6c,d). The visual representation of the experimental results is given in Figure 6e.

**Figure 7:**
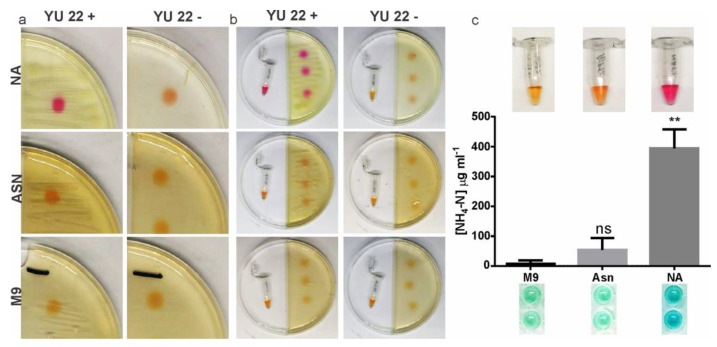
Evidence for the media alkalization by ammonification (direct and volatile) in *Kalamiella piersonii* YU22: (**a**) Six-days old nutrient agar (NA), L-asparagine agar (Asn) and M9 agar (M9) drop-coated with 5 µL phenol red to visualize alkalization (left panel, top to bottom); right panel, respective cell-free controls; (**b**) ammonification of liquid media placed in adjacent compartment was achieved through the emission of volatile NH_3_ by YU22 grown on NA, Asn and M9 agar plates. (**c**) Colorimetric determination of NH_4_-N trapped in liquid media; upper panel shows the pH change with phenol red indicator and lower panel, the sections of the plate showing the intensity of ammonia produced. YU22+, with cells; YU22-, without cells. Data points are mean ± SD (*n* = 4). ** *p* < 0.01, ns: not significant compared to M9.

Amino acid-driven ammonification is an energy-independent process, and we predicted its occurrence in solid and liquid culture media pre-inoculated with YU22. The volatile nature of ammonia and its capacity to drive media pH to alkaline range were two important factors exploited to screen possible ammonification reaction of YU22 in the solid and liquid media. The biotic (YU22) and abiotic (NH_3_ aqueous) assay for capturing biogenic ammonia or aqueous NH_4_ was tested using partition plate assay that showed confirmatory results (Figure 7a–c). Long term (6 days) growth of YU22 in nutrient agar plates showed strong local alkalization and released volatile ammonia. Similarly, moderate ammonification in solid and liquid culture was detected in M9 media containing asparagine.

### 2.6. Growth in Synthetic Urine and Struvite Crystallization In Vitro Mediated by YU22

**Figure 8:**
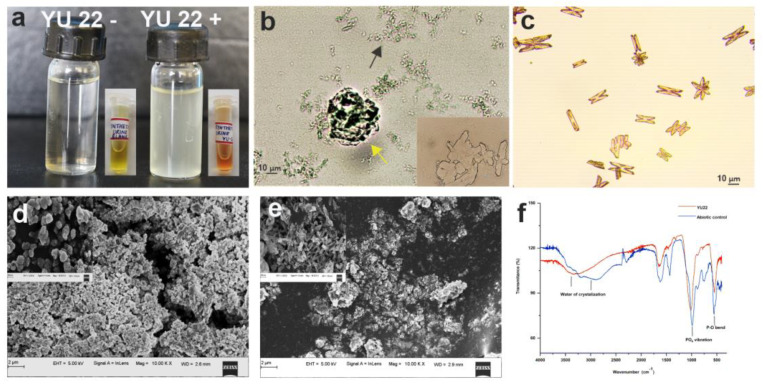
Growth in synthetic urine and promotion of struvite crystallization by *Kalamiella piersonii* YU22: (**a**) YU22 grown in synthetic urine (YU22+) and synthetic urine without bacteria (YU22-) and the adjacent Eppendorf tubes showing pH change with phenol red indicator, (**b**) light microscopic images of biogenically (YU22) produced struvite crystals in the synthetic urine. Inset showing the crystals at 100 X magnification, yellow arrow showing aggregated crystal and black showing smaller crystals, (**c**) abiotically produced struvite crystals by the addition of aq. ammonia to synthetic urine, (**d**,**e**) SEM of the biogenic and abiotically produced crystals respectively with insets showing the crystal interiors and (**f**) FT-IR spectra of the biogenic and abiotically produced crystals showing characteristic peaks for struvite.

Based on the evidence on the media alkalization and amino acid breakdown releasing ammonia to the local environment, bacterial growth in synthetic urine was assessed by supplementing 0.1% peptone. The results showed a similar trend in the bacterial growth and media pH (Figure 8a). Further, in vitro experiments to study the possible role of YU22 in struvite crystallization showed formation of crystals in the synthetic urine media. During the early phase of incubation, small crystals were formed that continued to grow to >10 µ in size with increasing growth and pH. At 36 h of incubation, crystal aggregates were observed having distinct crystal morphology and the microscopy revealed the viable and highly motile bacteria around the crystals (Figure 8b). In the abiotic set, struvite crystals were formed in the synthetic urine by the addition of aq. ammonia solution. Here, the crystallization took place rapidly within a few minutes at a pH of 8.0 and displayed typical twin crystal morphology (Figure 8c). These crystals subjected to SEM after drying showed intricate structure of the crystallite aggregates (Figure 8d,e). The crystals were identified based on the FT-IR spectral analysis. Both biogenic (YU22) and the abiotic samples showed similar FT-IR spectral peaks characteristic of struvite with signature peaks (Table 1, Figure 8f).

### 2.7. Antibiotic Susceptibility Pattern

The antibiotic susceptibility pattern of YU22 against the tested antibiotics is shown in Table 2. The strain YU22 was sensitive to many antibiotics and resistance to a few antibiotics such as ampicillin, ceftazidime, penicillin, vancomycin, piperacillin and rifampicin. The genome data showed several genes associated with antibiotic resistance and efflux pumps similar to the type strain as previously explained (Table S3).

## 3. Discussion

The genomic analysis of *K. piersonii* IIIF1SW-P2^T^ and strain YU22 showed similar genome structure sharing 98.9% OrthoANI value. The type strain *K. piersonii* IIIF1SW-P2^T^ has been already reported to possess many genes involved in the virulence and antibiotic resistance [13]. Based on the computational analysis (Pathogen Finder), the ISS isolate was considered to be non-pathogenic, in spite of the presence of virulence genes in the genome. We carried out a detailed investigation on the strain YU22 as this strain was a clinical isolate harbouring multiple genes for virulence. The antibiotic resistance patterns of the type strain and the strain YU22 were similar with the latter being sensitive to most of the Gram-negative antibiotics. To be pathogenic, the bacteria should express virulence factors to invade and metabolic adaptations to survive in the host environment. Here we discuss three aspects of *K. piersonii* strain YU22: (i) the virulence factors to establish its pathogenicity, (ii) metabolic capabilities for urinary tract colonization and (iii) role in struvite stone formation at least in the early crystallization process.

**Figure 9:**
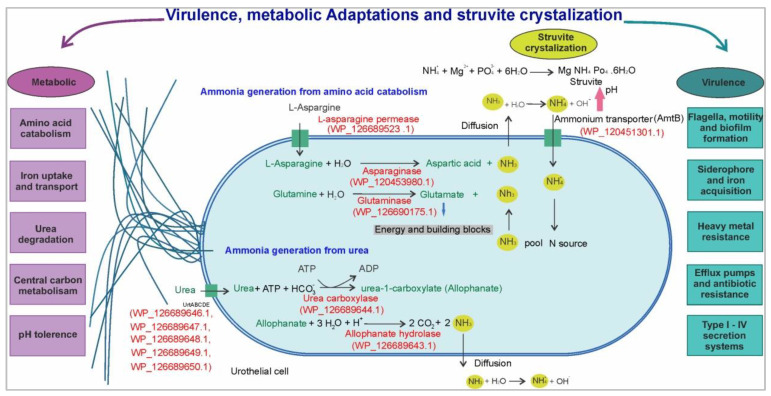
Schematic representation of the urea and amino acid breakdown pathways present in *Kalamiella piersonii* YU22: Ammonia production from urea is achieved by two distinct enzymes in which urea carboxylase (UC) is ATP dependent. The ammonia generation from amino acid breakdown is an energy independent mechanism involving enzymatic hydrolysis of amino acids. The ammonia released is converted to ammonium ions increasing the pH. Ammonium ions under increased pH interact with phosphate and magnesium ions forming struvite crystallization. The adaptation to urinary tract, invasion, colonization and survival of YU22 is possible due to presence of virulence factors, motility and metabolic capabilities to survive in nutrient limited urine.

*K. piersonii* strain YU22, a urine isolate, has shown several virulence factors and metabolic characteristics that could promote colonization of the urinary tract and formation of struvite stones through release of ammonia and alkalization of the urine. This is shown schematically in Figure 9.

The genomic and experimental evidence reveals the virulence factors needed for successful host cell colonization by *K. piersonii* strain YU 22. The strong adherence factors and motility are important virulence factors along with the iron acquisition siderophore system in YU22. It displayed strong swimming and swarming motilities that are commonly associated with many uropathogens. Swarming motility is flagellum dependent migration across a surface unlike swimming motility, which is movement in the liquid medium. For example, *Proteus mirabilis* has a strong swarming motility and initiates swarming in response to specific nutrients and environmental cues that are present in normal human urine [19]. In uropathogenic *Escherichia coli* (UPEC), swarming motility contributes to the virulence by enabling it to disseminate into the urinary tract, to escape host immune responses, and to disperse to new sites within the urinary tract [20]. Swarming motility and biofilm formation are population dependent and controlled by quorum sensing. Presence of quorum sensing system is identified in the genome of both YU22 and the type strain *K. piersonii* IIIF1SW-P2T.

Siderophores, the iron-binding factors, allow the bacteria to compete with the host for iron bound to haemoglobin, transferrin and lactoferrin. Uropathogenic *Pseudomonas aeruginosa*, *Klebsiella pneumoniae*, *E. coli* and *Yersinia* spp. utilize the siderophore for iron acquisition to promote intracellular colonization within the iron-limiting urine and bladder epithelial cells. Siderophores also have deeper role in virulence beyond simple iron chelation, inducing mitophagy, hypoxic responses, activating transcriptional pathways, affecting cellular behaviour and cytokine production [21].

Among other molecular features that a bacterium needs to infect and survive inside its host are two components secretion systems (I to IV type) and antibiotic resistance genes. From the genome sequence data, many antibiotic resistant genes were identified, and among them, the polymixin resistance genes (*pmrA*, *pmrB* and *pmrC*) were found in YU22 having high similarity with *K. perirsonii* IIIF1SW-P2T. The genes encoding for β-lactamase (EC-3.5.2.6), fusaric acid resistance cluster and bacitracin resistance in addition to efflux pumps are also identified. Among the efflux pumps, the resistance-nodulation-cell division (RND), outer membrane channel protein (OMP), outer membrane factor (OMF), major facilitator superfamily (MFS) and membrane fusion protein (MFP) family genes are the significant ones found in YU22. The tripartite multidrug efflux systems present are RND type MdtABC-TolC, RND type AcrAB-TolC and MFS type EmrKY-TolC and EmrAB-TolC. The genes involved in the metal resistance are well explained for the type strain *K. perirsonii* IIIF1SW-P2T, and the same is found in YU22 genome. Metal resistance is also one of important mechanisms pathogens employ to overcome the host cell defences.

The in vitro cell infection studies showed that YU22 was successful in cell attachment, and during the infection process there was a significant cell death. To establish an infection, a bacterium must adhere to the cells and multiply. To achieve this, YU22 has the attachment mechanisms, flagella, lipopolysaccharides and secretions systems as identified from the genome sequencing and that are also common to other uropathogens.

Second important feature of an uropathogen, is the metabolic adaptations to live in the nutrient restricted urine and to successfully counter the host defence. The microenvironment of the urinary tract is challenging and diverse with a constant flux of highly concentrated excretory products released due to the metabolic activity. However, uropathogens that colonize the urinary tract have the ability to utilize these conditions by numerous nutritional and physiological adaptations for survival in the niche [9,22,23,24]. Among the metabolic features, the ability to utilize broad range of nitrogen compounds including urea is the key factor. The essential genes *ureA*, *ureB* and *ureC* encoding the functional urease are absent in the genome of YU22 and IIIF1SW-P2T; however, the genes for uptake of urea, *urtABCDE*, are present.

Degradation of urea into ammonia can be achieved by two different enzymatic pathways mediated by the enzyme urease and urea amidolyase. The latter is a two-step enzyme system with urea carboxylase (EC 6.3.4.6) and allophanate hydrolase (EC 3.5.1.54). YU22 utilizes the alternative pathway comprising urea carboxylase and allophanate hydrolase enzymes for urea degradation. The genes involved in YU22 showed high sequence similarity (>99%) with the *K. perisonii* IIIF1SW-P2T. The strain YU22 was able to grow in urea containing media however required other carbon sources such as glucose in low concentration for the abundant growth. Change in the media pH in YU22 culture grown in urea can be attributed to the urea utilization. In *Candida albicans* and other fungi, the urea carboxylase and allophanate hydrolase activities are contained within a single polypeptide chain whereas in bacteria, these exist as two physically distinct ones, showing their critical role in the urea degradation pathway as functional enzymes. The crystal structure and functional analysis of a UPEC specific protein, c4763, a homologue of allophanate hydrolase, showed its functional role in urea breakdown, replacing the urease function and serving as a fitness factor for the bacteria [25]. Similarly, the urea amidolyase activities in *C. albicans* contribute to virulence and kidney pathogenesis by eliciting inflammatory response in host cells [26]. It has been postulated that the selective advantage of using urea amidolyase over urease (e.g., in *Saccharomycetes* species) is to evade all Ni^2+^ and Co^2+^ dependent metabolisms [18]. Further, there is also great interest on the evolutionary significance of these enzymes. The phylogenetic analysis of UALs and related proteins showed distinct phyletic position occupied by the tested strains besides substantiating the evolutionary origin of the fungal UALs and UC through the horizontal gene transfer as put forth by Strope et al. [18]. YU22 yielded no significant cell growth in the presence of urea despite having a two-step urea metabolizing enzyme system suggesting an alternative mechanism of ammoniagenesis. Moreover, further detailed investigations on the urea metabolism and gene expression patterns on prolonged incubation conditions remain to be undertaken to establish the role of UC and AH enzymes.

The other nitrogen sources available in the urine are the pool of amino acids and small peptides that serve as carbon sources for the bacteria residing in the urinary tract. [9,22]. Genetic and metabolic adaptation studies of UPEC have shown that amino acids and peptides serve as the major source of carbon during the UTI [23]. Apart from the two amino acids tested, namely, L-asparagine and L-glutamine, the genes for other amino acid breakdown were also found suggesting the metabolic versatility of the strain YU22. In the nutrient broth containing peptone, bacteria grew luxuriously and changed the pH of the media to highly alkaline (pH~8.5). Similar results were observed in the synthetic urine supplemented with peptone. Previous studies have also provided evidence from in vitro studies that peptone influences the growth and pH of the synthetic urine and modulates the lithogenesis in a lower pH than needed under normal conditions [27]. The bacterium was tolerant to wide range of pH (5–10) giving advantages for its survival in the fluctuating urine ecosystem. These findings suggest that YU22 is capable of utilizing both amino acids and small peptides effectively as carbon source. Degradation of amino acid (D-serine) by *P. mirabilis* provides fitness advantage in the urinary tract during infection [28]. Volatile ammonia released by *P. mirabilis* affects the survival of both planktonic and biofilm cultures of *K. pneumoniae* and exert bactericidal effect on *E. coli* showing that ammonia is also involved in this inter-species competition in the urinary tract [29]. The most highly evolved or adapted pathogens are the ones that acquire necessary nutritional substances for growth and dissemination with the smallest expenditure of energy and least damage to the host.

Having explored the virulence factors and the metabolic capabilities of YU22 that are needed for invasion and colonization in the urinary tract, our final hypothesis was to test whether strain YU22 could play a role in struvite stone formation although it lacked the enzyme urease. As far the current understanding goes, for struvite stone formation, presence of the enzyme urease is the most essential and has been established by many studies. UTI due to urease positive uropathogens such as *Proteus mirabilis*, *Klebsiella* spp., *Proteus* spp., *Providencia* spp., *Morgenalla morganii* spp. and *Serratia ureilytica* have been implicated in struvite stone disease [30,31]. However, there are arguments on the role of urease negative organisms in struvite crystallization. Moreover, the process of urease-induced crystallization can be influenced by urease-negative strains, and an infection with urease-producing microorganisms is not obligatory for the formation of this type of stones [32,33]. Urease negative bacteria such as *Enterococcus* spp., *E. coli*, *Streptococcus* spp., *Citrobacter* spp., *Peptostreptococcus* spp. and *Bacteroides fragilis* are commonly identified in the urine of struvite stone patients [5,34].

The mechanism of struvite stone formation involves the breakdown of urea by urease into ammonia and carbon dioxide, creating alkaline urine (pH 7.2–8.0), and this pH favours the crystallization of magnesium ammonium phosphate [3,35]. A few studies have proposed that struvite formation by microorganisms results from the release of ammonium either by decomposition of organic material or by hydrolysis of urea by urease by the microorganism resulting in the accumulation of ammonium ions in urine [32,36]. However, there is limited evidence of this [27]. Our experimental results provide further evidence of the production of ammonia and media alkalization by YU22 by breaking down amino acids and peptides. The ammonia is converted to ammonium ions in the aqueous media increasing the pH of the media/synthetic urine. In the process of struvite crystallization, nucleation and crystal growth are the two important steps. Nucleation occurs when the three ions (Mg^2+^, NH_4_^+^ and PO_4_^3−^) that are influenced by the pH combine, and when saturation of these ions occurs. The nucleation process is followed by the crystal growth phase, in which the ions in the solution/media are deposited in the nucleus, and the aggregation of the crystals takes place. This process has taken place during the struvite crystallization in vitro in the synthetic urine mediated by the bacterium YU22. Further extensive studies on urease negative uropathogens may expose the multiple processes involved in the struvite lithogenesis.

Based on the literature and our experimental results, it can be proposed that urease negative bacteria associated with struvite stones having urea carboxylase and allophanate enzyme activity or capability to utilize amino acids or small peptides and other nitrogenous compounds present as the excretory products may result in release of ammonia and alkalization of the urine. This condition will be similar to the urease induced struvite crystallization, wherein the ammonium ions increase the pH of the urine that favours the struvite crystallization. The conditions may also depend on the central carbohydrate metabolism pathways. The speed at which the crystal growth takes place may differ from urea splitting process. The formation of struvite crystal initiated due to peptide/amino acid breakdown by urease negative bacteria may serve as a nidus and attract other minerals forming more complex mixed stone complex. Together with the data from whole genome analysis and the validation experiments, we provide evidence for considering *K. peirsonii* strain YU22 as an uropathogen. We also have provided further evidence to suggest that ammonia produced from peptide/amino acid breakdown can also have a significant role in the formation and crystallization of struvite by *K. peirsonii* strain YU22 and other urease negative uropathogens. Further studies on other isolates of *K. peirsonii* are warranted to assess the health risks.

## 4. Methodology

### 4.1. Patient Sample and Bacterial Isolation

The strain YU22 was isolated from the urine of a patient with symptomatic kidney stone disease reported from the Department of Urology in a Tertiary Care Hospital. All the procedures involving human participants were carried out following the rules of the Declaration of Helsenki and were approved by the Institutional Ethics Committee of Yenepoya University, Mangalore, India (YUEC.022/16), and the Institutional Scientific Review Board (YRCSRB034/17). Written informed consent was obtained from the participants before collecting the urine and kidney stone samples. The details of basic demographics and the clinical investigations such as kidney function test, urinalysis, complete blood count and stone mineralogy were obtained from the medical records. For urine culture, the first early morning urine sample (clean catch specimen, approx. 30 mL) was collected before administration of any medications. The urine was cultured using MacConkey agar, blood agar and nutrient agar (NA) (Hi-Media, Mumbai, India). The isolate designated as YU22 was maintained in NA plates and stored as glycerol stocks at minus 80 °C deep freezer.

### 4.2. Biochemical and Physiological Analysis

The strain YU22 was tested for its growth in Luria–Bertani (LB) agar, tryptic soy agar (TSA) and NA at 32°C. The morphological and biochemical characteristics and Gram staining were performed using routine microbiological methods. Cell growth at pH 5–10 (1.0-unit interval) was examined using the following buffer systems: 0.1 M citric acid/0.1 M trisodium citrate for pH 5.0; 0.2 M Na_2_HPO_4_/0.2 M NaH_2_PO_4_ for pH 6.0–8.0 and 0.1 M NaHCO_3_/0.1 M Na_2_CO_3_ for pH 9.0–10.0. To test the oxidation of sole carbons, cell suspension from overnight cultures was prepared in physiological saline and inoculated into Biolog GN2 microplate. Similarly, cell suspensions were also inoculated to API 20 E, API 20 NE, API ZYM and API 50CH (BioMérieux, Marcy-l’Étoile, France) strips for biochemical and enzymatic analysis. All these tests were performed according to manufacturer’s instructions, and the results were recorded within 24 h incubation at 32 °C.

### 4.3. 16S rRNA Gene Sequencing, Whole Genome Sequencing and Bioinformatics

The genomic DNA was isolated from the bacterial cells using QIAamp DNA Mini Kit (Qiagen Inc.). 16S rRNA gene was amplified using 3F/9R universal primer pair, 3F (5′-CCTACGGGAGGCAGCAG-3′) and 9R (5′-AAGGAGGTGATCCAACCGCA-3′) and sequenced for the identification of the strain. 16S rRNA gene sequence similarity values were computed using EzBiocloud server for the taxonomy [37]. The whole genome sequencing was carried out in an Illumina NextSeq 2500 platform using 2 × 250 paired-end libraries. The sequences obtained were quality processed and trimmed, and de novo assembly of the sequences was performed using SPAdes genome assembler (ver 3.10) [38]. FASTQC was used to assess the raw reads quality, and trimming was performed by trimmomatic (ver 0.35), identifying a phred cutoff of Q20. Assembled genome was annotated using NCBI Prokaryotic Genome Annotation Pipeline. Overall genome relatedness index, measured as the Orthologous average Nucleotide Identity (OrthoANI), was calculated using the OrthoANI application of EzBioCloud [39]. For this, 10 whole genomes of closely related taxon were used (Table S6).

Detailed genomic analysis was carried out to study the metabolic pathways, virulence genes and antibiotic resistance genes to get insights into the bacterial lifestyle inside the urinary tract. For automated annotation, the genome sequence was uploaded to the web annotation service Rapid Annotations using Subsystems Technology (RAST) [40]. The gene features of essential biosystems were further manually confirmed using BLASTp (https://blast.ncbi.nlm.nih.gov/Blast.cgi) against non-redundant database of National Center for Biotechnology Information (NCBI). Essential enzyme functional prediction was obtained from Kyoto Encyclopedia of Genes and Genomes (KEGG; http://www.genome.jp/kegg/) using KEGG Automatic Annotation server (KAAS) and UniProt (The UniProt Consortium). This functional annotation was used to reconstruct the metabolic pathways.

For the phylogeny of the genes involved in the key metabolic pathways, sequences were retrieved from NCBI and UniProt, aligned by Clustal_X [41] and analysed by MEGA-5 (Molecular Evolutionary Genetics Analysis, version 5.0) [42]. Maximum likelihood algorithm [43] was used for the phylogenetic analysis of urea carboxylase (UC) and allophanate hydrolase (AH). The tree topology was evaluated by using the bootstrap re-sampling method based on 1000 replications [44].

### 4.4. Investigation on the Virulence Factors in YU22

Based on the bioinformatics analysis, the important virulence factors such as motility, biofilm formation, DNase activity and production of siderophores were investigated using standard laboratory methods. The motility was tested by hanging drop method using a light microscope, and based on the directional movements, the motility was inferred. The swimming motility was tested using 0.3% agar [45] and swarming motility using 0.5% agar in LB base [46]. The swimming and swarming diameters were measured, and for visualization, photographs were recorded. The presence of flagella was assessed microscopically using flagellar staining technique [47]. Scanning electron microscopy was also used to demonstrate the cell morphology. Crystal violet staining method was used for estimating the biofilm formation [48]. Siderophore production was investigated using CAS agar plate that additionally contained 10 mM glucose, and formation of orange halo around the colonies on incubation was considered as positive for siderophore production [49].

### 4.5. In Vitro Assays for Host Cell Attachment and Cell Killing

To evaluate the bacterial interaction with the host cells, in vitro cell attachment assay was performed using human embryonic kidney cells (HEK293T). HEK293T cells were procured from National Centre for Cell Sciences (Pune, India), maintained in Dulbecco’s Modified Eagle Medium (DMEM) and supplemented with 10% FBS and 1% antibiotic-antimycotic solution at 37 °C in 5% CO_2_ atmosphere. For infection studies, HEK 293T cells were seeded onto 96-well plates (10,000 cells/well) in DMEM without the antibiotics and infected with the bacteria at a multiplicity of infection (MOI) of 10 for 3 h at 37 °C. Bacterial attachment to the HEK cells was estimated by the plate count method described earlier [50]. Briefly, the cells were washed with PBS, homogenized, serially diluted, and plated on NA. The CFU counts were recorded after 24 h incubation, and percentage cell attachment was calculated with respect to control. For estimation of cell killing effect, the cells were infected with YU22 for 4 h, and viable and dead cells were counted by trypan blue dye exclusion method using a cell counter (LUNA-II^TM^, Logos Biosystems, Anyang, South Korea). The cell killing was calculated with reference to un-infected cell control. To visualize the infected cells, live/dead staining was performed using acridine orange ethidium bromide dyes, and images were captured using a fluorescence imager (ZOE, Bio-Rad, Hercules, CA, USA). Giemsa staining was used to localize the bacteria in the infected cells using a light microscope.

### 4.6. Growth and Metabolic Activities of K. piersonii YU22 in Glucose, Urea, Amino Acids and in Urine

To study the effect of glucose, urea and the amino acids on the growth, metabolism and media alkalization, many growth experiments were performed. For the inoculum, the bacterial cells were cultured in NB for 16 h, and 20 µL cell suspension was inoculated in to 4 mL media taken in 20-mL glass test tubes. To monitor the growth in presence of glucose and urea, different concentrations (0–10 mM) of glucose solutions were prepared in 1X M9 (Sigma, St. Louis, MI, USA) media with and without 10 mM urea (JT Baker, Phillipsburg, NJ, USA) supplement. Subsequently, growth was tested in 1X M9 containing 10 mMglucose and/or 10 mM L-asparagine/10mMglutamine in the absence of urea. Finally, growth was tested in various concentrations (0, 0.625, 1.25, 2.50, 5 and 10 mM) of asparagine prepared in 1X M9 (Sigma, St. Louis, MI, USA) containing 10 mM urea and 0.625 Mm glucose. Cells were cultivated for 48 h at 32 °C (120 rpm). All culture experiments were performed in four biological repeats and optical density readings were recorded using a multimode plate reader at OD_600_. Similar experiments were performed to assess the growth and evolution of media pH in the synthetic urine and healthy human urine by inoculating the bacteria to the filter sterilized synthetic and healthy human urine, and incubation was done at 37 °C for 48 h.

#### 4.6.1. Colorimetric Determination of Metabolic Activity, Change in Media pH and NH_4_-N

The metabolic activity of the bacteria grown under different carbon sources was determined by adding 10% (*v*/*v*) Alamar blue reagent (Invitrogen, Carlsbad, CA, USA) to cell suspensions and recording the absorbance at 570 nm and 600 nm. Percentage dye reduction was calculated according to manufacturer’s instructions. Change in media pH was estimated by adding 10% (*v*/*v*) phenol red (Sigma, St. Louis, MI, USA) indicator solution to the cell-free supernatant in a 96-well microplate reader and recording the absorbance at 415 nm (acidity) and 560 nm (alkalinity) based on the absorbance maxima of the indicator colour under acidic and alkaline pH. The pH was also measured using a digital pH electrode.

The release of NH_4_-N was estimated according to Baethgen and Alley [51] with the slight modifications. Briefly, 20 µL of 10-fold-diluted cell-free supernatants were added to 110 µL working buffer solution in a 96-well microplate and mixed with 80 µL sodium salicylate-sodium nitroprusside solution (15%:0.03%) followed by 40 µL sodium hypochlorite (0.32%). The plates were incubated at 32 °C for 30 min and the absorbance was read at 650 nm using Asys UVM 340 (Biochrom, Holliston, MA, USA) micro-plate reader. NH_4_-N was calculated from the standard graph plotted using NH_4_-N standard [(NH_4_)_2_SO_4_]. To establish the media alkalization by ammonification (direct and volatile) in *K. piersonii*, YU22 was grown in different agar plates for six days.

#### 4.6.2. Partition Plate Assay for Abiotic and Biotic Volatile NH_3_ Emission

To visualize and estimate the NH_3_ emission, partition plate assay was used with bacteria (biotic) and ammonia (abiotic). For abiotic assay, 20 µL of aqueous ammonium solution (32%) was taken in a 500-µL microfuge tube and placed in one compartment of partition plate. Another 500-µL microfuge tube containing 100 µL nutrient broth (NB) was placed in the adjacent compartment. Plate was tightly sealed using an insulation tape. Diffusion of volatile NH_3_ on solid and liquid media was qualitatively determined after incubation (12 h, 32 °C) followed by the addition of phenol red solution to the solid compartment (agar) and microfuge tubes. Partition plates without volatile NH_3_ supply were treated as blank.

For biotic assay, YU22 cells were streaked separately on NA, M9 agar and M9 agar supplemented with 10 mM asparagine or 10 mM urea casted on one compartment. A 500-µL microfuge tube containing 100 µL of respective solid agar (NA/blank, M9, M9 + 10 Mm asparagine and M9 + 10 mM urea) was placed in the adjacent compartments. Plates were tightly sealed, and the diffusion of volatile NH_3_ was determined qualitatively after six-days of plate incubation at 32 °C. NH_4_-N trapped in the liquid M9 broth was estimated after appropriate dilution with reference to standard curve.

### 4.7. In Vitro Struvite Crystallization Assays

In vitro crystallization assay was performed using YU22 as described elsewhere [52]. The synthetic urine was prepared according to Griffith [35] with the following composition (g/L) CaCl_2_·2H_2_O, (0.651); MgCl_2_·6H_2_O, (0.651); NaCl, (4.6); Na_2_SO_4_, (2.3); KH_2_PO_4_, (2.8); KCl, (1.6); NH_4_Cl, (1.0); sodium citrate, (0.65); sodium oxalate, (0.02); urea, (25.0); and creatinine, (1.1) and pH was adjusted to 6.5–6.8. To trigger bacterial growth, synthetic urine was supplemented with peptone water (0.1%). Bacteria was inoculated to an initial density of 10^5^ cells/mL to 10 mL filter sterilized synthetic urine and incubated at 37 °C for 24–48 h. Samples were retrieved at regular intervals for monitoring the pH and observed under microscope for the presence of crystals. For harvesting the crystals, 50 mL synthetic urine cultures were prepared by incubating for 36 h. The culture was filtered using Whatman^®^ No. 3 filter paper (pore size, 6 µm), which allows the bacterial cells to pass through the pores while retaining the relatively larger crystals. The crystals retained in the filter paper were oven dried (55 °C) and subjected to ATR-FTIR analysis (IR Spirit, Shimadzu). The IR spectrum was interpreted using the standard absorption peaks for the mineral type based on the literature [53]. For comparison, abiotic control was prepared by adding aqueous ammonia (0.1 M) to synthetic urine that increased the pH and precipitated the phosphate as magnesium and ammonium phosphate. These samples were analysed by light microscopy and FT-IR spectroscopy. The dried crystal samples were subjected to scanning electron microscopy using routine sample preparation procedure.

### 4.8. Antibiogram of K. piersonii YU22

The antibiotic susceptibility pattern of YU22 was carried out according to Kirby–Bauer disc diffusion assay [54]. Briefly, Muller Hinton agar plates were inoculated with overnight cultures of bacteria using a sterile cotton swab to prepare a uniform lawn of bacteria. Standard antibiotic discs (HiMedia, Mumbai, India) were placed on the agar plates and incubated upright at 37 °C. After 24 h incubation, the clear zone of growth inhibition around the discs was measured and susceptibility was interpreted based on the CLSI guidelines for each of the antibiotic.

### 4.9. Data Analysis and Visualization Tools

All the laboratory experiments were conducted at least in triplicates unless specified. The data is presented as mean ± SD, and Graph-Pad Prism (version 6) was used for generating the graphs. The BLAST Ring Image Generator (BRIG) Server {via http://brig.sourceforge.net} was used to generate comparatives in the genome *K. piersonii* YU22 against type strain *K. piersonii* IIIF1SW-P2^T^ in closed circle model [55]. Protein coding genes were analysed by COG database [56] using COGsoft [57] and Pfam domains were predicted using NCBI Batch CD-Search Tool [58]. We obtained 4670 total number of COGs assignments under different classes additionally, using the RAST server version 2.0 [59].

### 4.10. Nucleotide Sequence Deposition and Data Availability

*K. piersonii* strain YU22 has been deposited at Microbial Culture Collection (MCC), National Center for Cell Science Pune, under the accession number MCC 3118. This whole-genome shotgun project has been deposited at DDBJ/EMBL/GenBank under the BioProject ID: PRJNA510491, raw-reads with SRA accession number: SRP256420 and Genome accession number: RYDU00000000. The version described in this paper is in the first version, RYDU00000000.1.

This genome description and metabolic predictive annotation is available to Rapid Annotation using Subsystem Technology (RAST) server with Genome ID: 2364647.6. The other databases having analysis report for YU22 strain include, JGI-Genomes Online Database (GOLD) with Analysis Project ID: Ga0438604, and Integrated Microbial Genomes and Microbiomes (IMG/M) system for *K. piersonii* strain YU22 genome is available under IMG Genome ID 2870892799. The 16S rRNA gene sequence of YU22 strain derived with the Sanger sequencing method is available in the GenBank under accession number: MH021677.

## 5. Conclusions

This is the first report on the association of *K. piersonii*, a member of *Erwiniaceae* with urine of a kidney stone patient. The urease negative *K. piersonii* strain YU22 displayed several features indicative of its urinary tract association and involvement in struvite crystallization. The role of alternate urea degradation pathway mediated by urea amidolyase and allophanate hydrolase enzymes needs further exploration to associate it to urea utilization and breakdown.

## Figures and Tables

**Table 1:** FT-IR spectral peaks for the struvite crystals formed by YU22 in comparison to the abiotic control.

Assignments	Reported IR Frequencies Wave Numbers (cm^−1^)	Observed IR Frequencies Wavenumbers (cm^−1^)	
		Biotic (YU22)	Abiotic
**Absorption peak due to water of crystallization**			
H-O-H stretching vibrations of H_2_O of crystallization	3280 to 3550	3316	3550
H-O-H stretching vibrations of cluster of water molecules of crystallization	2060 to 2460	2206	2298
H-O-H bending modes of vibrations	1590 to 1650	1652	1622
Vagging modes of vibration of coordinated water	808	778	808
**Absorption peak due to PO_4_ units**			
V_1_ symmetric stretching vibration	930 to 995	995	988
V_2_ symmetric bending vibration	404 to 470	471	404, 455
V_3_ asymmetric bending vibration	1017 to 1163	1015, 1018	1018, 1164
V_4_ asymmetric bending modes	509 to 554	544	510, 555
**Metal-oxygen bonds**			
Metal-oxygen bonds	400–650	460	651
Deformation of OH linked to Mg^2+^	847	847	847

**Table 2:** Antibiotic sensitivity pattern of *K. piersonii* YU22.

Antibiotic	Sensitivity	Antibiotic	Sensitivity
Levofloxacin (5 µg)	S	Collistin (10 µg)	S
Merophenem (10 µg)	S	Tetracycline (30 µg)	S
Imipenem (10 µg)	S	Aztreonam (30 µg)	I
Co-trimoxozole (25 µg)	S	Ampicillin (10 µg)	R
Tobramycin (10 µg)	S	Ceftazidime (30 µg)	R
Ciprofloxacin (5 µg)	S	Penicillin–G (10 units)	R
Clindamycin (10 µg)	S	Vancomycin (30 µg)	R
Amikacin (30 µg)	S	Piperacillin (100 µg)	R
Gentamicin (10 µg)	S	Rifampicin (5 µg)	R

S: Sensitive, I: Intermediate and R: Resistant based on the zone of inhibition tested by Kirby Bauer disc diffusion method.

## Supplementary Materials

The following are available online at https://www.mdpi.com/2076-0817/9/9/711/s1. Supplementary Document S1: Summary of API 20 E, API 20 NE, API ZYM and API 50CH, Biolog GN2 assay results for *K. piersonii* YU22. Figure S1: Colony and cell morphology of *K. piersonii* YU22. (a) YU22 colonies growing on nutrient agar plates incubated for 24 h. (b) Scanning electron micrograph showing the size as 1 micron (scale bar 1 micron). Figure S2: Evolutionary relation between urea metabolizing enzymes. Maximum likelihood phylogenetic tree showing the evolutionary relation between urea metabolizing enzymes (two-step; urea carboxylase and allophanate hydrolase) found in *K. piersonii* YU22 to urea amidolyase (single-step) of fungi (shown in green). Bootstrap values (>70%) based on 1000 replications are shown at the nodes. The accession number is shown in parentheses. Bar, 0.5 substitutions per nucleotide position. UC, urea carboxylase; UAL, urea amidolyase; AH, allophanate hydrolase; *type strain of *K. piersonii* IIIF1SW-P2^T^. Yellow highlight indicates sole bacterium that harbour UAL. Table S1. Clinical investigation of urine and blood samples of the kidney stone patient: Table S2: 16S rRNA gene sequence analysis at EzBiocloud database showing closely related species of YU22 from *Erwiniaceae* (Data as of February 25, 2020). Table S3. Functional categories found in *K. piersonii* YU22 genome. Data extracted from Clusters of Orthologous *Genes* (*COG*) database COGsoft. Table S4. Virulence genes identified in the genome of *K. piersonii* YU22: motility, Antimicrobial resistance (AMR) multidrug efflux pumps, iron uptake and transport genes and heavy metal resistance genes. Table S5: Genes involved in urea cycle identified from the genome of *K. piersonii* YU22. Table S6: Details of the 10 taxon used for calculation of OrthoANI values.
