# Supplementary material for: First Report of Pathogenic Bacterium Kalamiella piersonii Isolated from Urine of a Kidney Stone Patient: Draft Genome and Evidence for Role in Struvite Crystallization
Source: Pathogens. 2020 Aug 29;9(9):711. doi: 10.3390/pathogens9090711 (PMC7558591; doi:10.3390/pathogens9090711)
Supplement: Supplementary file 1 [file pathogens-09-00711-s001.zip › Document S1.docx]

**Supplementary Material**

**Summary of API 20 E, API 20 NE, API ZYM and API 50CH, Biolog GN2 assay**

**Positive results**

**API20E**

In API 20 E strip, positive for the hydrolysis of *o*-nitrophenyl-*β-*D-galactopyranoside, acetoin production, and fermentation/oxidation of D-glucose, D-mannitol, inositol, L-rhamnose, D-melibiose and L-arabinose.

**API 20 NE**

In API 20 NE strip, positive for nitrate reduction, fermentation of D-glucose, hydrolysis of esculin and *p*-nitrophenyl-*β-*D-galactopyranoside, and assimilation of D-glucose, L-arabinose, D-mannose, D-mannitol, *N*-acetyl-glucosamine, D-maltose, potassium gluconate, malic acid and trisodium citrate.

**API ZYM**

In API ZYM test strip, positive for the activities of alkaline phosphatase, esterase (C 4), esterase lipase (C 8), leucine arylamidase, valine arylamidase, cystine arylamidase, acid phosphatase, naphthol-AS-BI-phosphohydrolase, *β*-galactosidase and *β*-glucosidase.

**API 50 CH strip**

In API 50 CH strip, positive for acid production from glycerol, L-arabinose, D-ribose, D-xylose, D-galactose, D-glucose, D-fructose, D-mannose, L-rhamnose, inositol, D-mannitol, methyl-*α-*D-mannopyranoside, methyl-*α-*D-glucopyranoside, *N*-acetyl-glucosamine, esculin ferric citrate, salicin, D-cellobiose, D-maltose, D-lactose, D-melibiose, D-trehalose, xylitol, D-lyxose and L-fucose.

**Biolog GN2**

In the Biolog GN2 microplate, positive for the oxidation of *α*-cyclodextrin, dextrin, glycogen, Tween 40, Tween 80, *N*-acetyl-D-galactosamine, *N*-acetyl-D-glucosamine, adonitol, L-arabinose, D-arabitol, D-cellobiose, D-fructose, L-fucose, D-galactose, gentiobiose, *α*-D-glucose, m-inositol, *α*-D-lactose, lactulose, maltose, D-mannitol, D-mannose, D-melibiose, *β*-methyl-D-glucoside, D-psicose, D-raffinose, L-rhamnose, D-sorbitol, sucrose, D-trehalose, turanose, xylitol, pyruvic acid methyl ester, succinic acid mono-methyl ester, acetic acid, *cis*-aconitic acid, citric acid, formic acid, D-galactonic acid lactone, D-galacturonic acid, D-gluconic acid, D-glucosaminic acid, D-glucuronic acid, *α*-hydroxybutyric acid, *β*-hydroxybutyric acid, *γ*-hydroxybutyric acid, *p*-hydroxy phenylacetic acid, itaconic acid, *α*-keto glutaric acid, D,L-lactic acid, malonic acid, propionic acid, quinic acid, D-saccharic acid, sebacic acid, succinic acid, bromosuccinic acid, succinamic acid, glucuronamide, L-alaninamide, D-alanine, L-alanine, L-alanyl glycine, L-asparagine, L-aspartic acid, L-glutamic acid, glycyl-L-aspartic acid, glycyl-L-glutamic acid, L-histidine, hydroxy-L-proline, L-leucine, L-ornithine, L-phenylalanine, L-proline, L-pyroglutamic acid, D-serine, L-serine, L-threonine, D, L-carnitine, *γ*-amino butyric acid, urocanic acid, inosine, uridine, thymidine, phenyethylamine, putrescine, 2-aminoethanol, 2,3-butanediol, glycerol, D,L-*α*-glycerol phosphate, *α*-D-glucose-1-phosphate and D-glucose-6-phosphate.

**Negative results**

**API 20 E**

In API 20 E, negative for the activities of arginine dihydrolase, lysine decarboxylase, ornithine decarboxylase, urease, tryptophane deaminase and gelatinase, citrate utilization, production of H_2_S and indole, and fermentation/oxidation of D-sorbitol, D-sucrose and amygdalin.

**API 20 NE**

In API 20 NE test strip, negative for indole production, arginine dihydrolase and urease activities, hydrolysis of gelatin, and assimilation of capric acid, adipic acid and phenylacetic acid.

**API ZYM**

In API ZYM test strip, negative for lipase (C 14), trypsin, *α*-chymotrypsin, *α*-galactosidase, *β*-glucuronidase, *α*-glucosidase, *N*-acetyl-*β*-glucosaminidase, *α*-mannosidase and *α*-fucosidase.

**API 50 CH**

In API 50 CH strip, negative for acid production from erythritol, D-arabinose, L-xylose, D-adonitol, methyl-*β-*D-xylopyranoside, L-sorbose, dulcitol, D-sorbitol, amygdalin, arbutin, D-saccharose, inulin, D-melezitose, D-raffinose, amidon, glycogen, gentiobiose, D-turanose, D-tagatose, D-fucose, D-arabitol, L-arabitol, potassium gluconate, potassium 2-ketogluconate and potassium 5-ketogluconate.

**Biolog GN2**

In Biolog GN2 microplate, negative for the oxidation of i-erythritol, *α*-keto butyric acid and *α*-keto valeric acid.
