# Supplementary material for: First Report of Pathogenic Bacterium Kalamiella piersonii Isolated from Urine of a Kidney Stone Patient: Draft Genome and Evidence for Role in Struvite Crystallization
Source: Pathogens. 2020 Aug 29;9(9):711. doi: 10.3390/pathogens9090711 (PMC7558591; doi:10.3390/pathogens9090711)
Supplement: Supplementary file 1 [file pathogens-09-00711-s001.zip › Table S1.docx]

**S2 Table.**  **Clinical investigation of urine and blood samples of the kidney stone patient**

| **Parameter (units)** | **Value** |
| --- | --- |
| Haemoglobin (g/dL) | 13.8 |
| RBC (millions/µL) | 4.57 |
| PCV | 41 |
| MCV (fl) | 90.2 |
| MCH (pg) | 30.2 |
| MCHC (g/L) | 33.5 |
| Serum calcium (mg/dL) | 10 |
| Uric acid (mg/dL) | 7.9 |
| Random blood sugar (mg/dL) | 86 |
| Blood urea (mg/dL) | 20 |
| Serum creatinine (mg/dL) | 1.4 |
| ESR (mm/h) | 35 |
| Na (mmol/L) | 143 |
| K (mmol/L) | 3.1 |
| Cl (mmol/L) | 99 |
| Urine-colour | Pale yellow |
| Specific gravity | 1.02 |
| Urine pH | ~7 |
| Albumin | Negative |
| Haemoglobin | Absent |
| RBC | Nil |
| Pus (/HPF) | 1 to 2 |
| Epithelial cell | 1 to 2 |
| Casts | Nil |
| Urobilinogen | Normal |
| Ketone bodies | Negative |
| Bilirubin | Negative |
| Nitrite | Negative |
