# Supplementary material for: First Report of Pathogenic Bacterium Kalamiella piersonii Isolated from Urine of a Kidney Stone Patient: Draft Genome and Evidence for Role in Struvite Crystallization
Source: Pathogens. 2020 Aug 29;9(9):711. doi: 10.3390/pathogens9090711 (PMC7558591; doi:10.3390/pathogens9090711)
Supplement: Supplementary file 1 [file pathogens-09-00711-s001.zip › Table S2.docx]

**Table S3.** **16S rRNA gene sequence analysis at EzBiocloud database showing closely related species of YU22 from *Erwiniaceae*** (Data as of Feb 25^th^ 2020).

| **Rank** | **Name** | **Strain** | **Authors** | **Genus** | **Accession No** | **Similarity (%)** |
| --- | --- | --- | --- | --- | --- | --- |
| 1 | *Kalamiella piersonii* | IIIF1SW-P2 | Singh et al. 2019 | *Kalamiella* | RARB01000003 | 100 |
| 2 | *Pantoea cypripedii* | LMG 2657 | Hori 1911  Brady et al. 2010 | *Pantoea* | MLJI01000002 | 97.88 |
| 3 | *Pantoea sp* | AS-PWVM4 | Khatri et al. 2013 | *Pantoea* | ASZC01000041 | 97.81 |
| 4 | *Flavobacterium acidificum* | LMG 8364 | Steinhaus 1941 | *Pantoea* | JX986959 | 97.56 |
| 5 | *Pantoea allii* | LMG 24248 | Brady et al. 2011 | *Pantoea* | AY530795 | 97.47 |
| 6 | *Pantoea beijingensis* | LMG 27579 | Liu et al. 2013 | *Pantoea* | KC846071 | 97.44 |
| 7 | *Pantoea anthophila* | LMG 2558 | Brady et al. 2009 | *Pantoea* | EF688010 | 97.43 |
| 8 | *Pantoea hericii* | JZB 2120024 | Rong et al. 2016 | *Pantoea* | KU189725 | 97.34 |
| 9 | *Pantoea vagans* | LMG 24199 | Brady et al. 2009 | *Pantoea* | EF688012 | 97.34 |
| 10 | *Pantoea ananatis* | LMG 2665 | Serrano 1928  Mergaert et al. 1993 | *Pantoea* | JMJJ01000010 | 97.33 |
| 11 | *Pantoea septica* | LMG 5345 | Brady et al. 2010 | *Pantoea* | MLJJ01000077 | 97.33 |
| 12 | *Pantoea brenneri* | LMG 5343 | Brady et al. 2010 | *Pantoea* | MIEI01000169 | 97.33 |
| 13 | *Erwinia mediterraneensis* | Marseille-P5165 | Ndiaye et al. 2019 | *Erwinia* | LR026978 | 97.33 |
| 14 | *Pantoea sp* | RIT388 | - | *Pantoea* | RMVG01000054 | 97.26 |
| 15 | *Mixta intestinalis* | 29Y89B | Prakash et al. 2015  Palmer et al. 2018 | *Mixta* | KP326384 | 97.20 |
