# Supplementary material for: First Report of Pathogenic Bacterium Kalamiella piersonii Isolated from Urine of a Kidney Stone Patient: Draft Genome and Evidence for Role in Struvite Crystallization
Source: Pathogens. 2020 Aug 29;9(9):711. doi: 10.3390/pathogens9090711 (PMC7558591; doi:10.3390/pathogens9090711)
Supplement: Supplementary file 1 [file pathogens-09-00711-s001.zip › Table S3.docx]

**Table S4**. **Functional categories found in *Kalamiella piersonii* YU22 genome**. Data extracted from Clusters of Orthologous Genes (COG) database COGsoft

| **Sl. No** | **Functional Category** | **No. of genes** |
| --- | --- | --- |
| 1 | Translation, ribosomal structure and biogenesis | 283 |
| 2 | RNA processing and modification | 1 |
| 3 | Transcription | 390 |
| 4 | Replication, recombination and repair | 160 |
| 5 | Chromatin structure and dynamics | 1 |
| 6 | Cell cycle control, cell division, chromosome partitioning | 55 |
| 7 | Nuclear structure | 0 |
| 8 | Defense mechanisms | 89 |
| 9 | Signal transduction mechanisms | 247 |
| 10 | Cell wall/membrane/envelope biogenesis | 310 |
| 11 | Cell motility | 114 |
| 12 | Cytoskeleton | 1 |
| 13 | Extracellular structures | 23 |
| 14 | Intracellular trafficking, secretion, and vesicular transport | 87 |
| 15 | Posttranslational modification, protein turnover, chaperones | 159 |
| 16 | Mobilome: prophages, transposons | 82 |
| 17 | Energy production and conversion | 234 |
| 18 | Carbohydrate transport and metabolism | 436 |
| 19 | Amino acid transport and metabolism | 451 |
| 20 | Nucleotide transport and metabolism | 111 |
| 21 | Coenzyme transport and metabolism | 217 |
| 22 | Lipid transport and metabolism | 172 |
| 23 | Inorganic ion transport and metabolism | 304 |
| 24 | Secondary metabolites biosynthesis, transport and catabolism | 103 |
| 25 | General function prediction only | 411 |
| 26 | Function unknown | 229 |
