# Supplementary material for: First Report of Pathogenic Bacterium Kalamiella piersonii Isolated from Urine of a Kidney Stone Patient: Draft Genome and Evidence for Role in Struvite Crystallization
Source: Pathogens. 2020 Aug 29;9(9):711. doi: 10.3390/pathogens9090711 (PMC7558591; doi:10.3390/pathogens9090711)
Supplement: Supplementary file 1 [file pathogens-09-00711-s001.zip › Table S4.docx]

**S5 Table: Virulence genes identified in the genome of *Kalamiella piersonii* YU22**.

(A). Motility

| **PATRIC ID** | **NCBI ID** | **Refseq ID** | ***Gene*** | **Product** |
| --- | --- | --- | --- | --- |
| **Motility and Chemotaxis** | |  |  |  |
| fig\|2497684.3.peg.1481 | RTY59975.1 | WP_120452005.1 | *flgN* | Flagellar biosynthesis protein |
| fig\|2497684.3.peg.1482 | RTY59976.1 | WP_120452007.1 | *flgM* | Negative regulator of flagellin synthesis (anti-sigma28) |
| fig\|2497684.3.peg.1483 | RTY59977.1 | WP_126688701.1 | *flgA* | Flagellar basal-body P-ring formation protein |
| fig\|2497684.3.peg.1484 | RTY59978.1 | WP_120452011.1 | *flgB* | Flagellar basal-body rod protein |
| fig\|2497684.3.peg.1485 | RTY59979.1 | WP_033789486.1 | *flgC* | Flagellar basal-body rod protein |
| fig\|2497684.3.peg.1486 | RTY59980.1 | WP_120452013.1 | *flgD* | Flagellar basal-body rod modification protein |
| fig\|2497684.3.peg.1487 | RTY59981.1 | WP_120452015.1 | *flgE* | Flagellar hook protein |
| fig\|2497684.3.peg.1488 | RTY59982.1 | WP_120452017.1 | *flgG* | Flagellar basal-body rod protein |
| fig\|2497684.3.peg.1489 | RTY59983.1 | WP_120452019.1 | *flgG* | Flagellar basal-body rod protein |
| fig\|2497684.3.peg.1490 | RTY59984.1 | WP_126688702.1 | *flgH* | Flagellar L-ring protein |
| fig\|2497684.3.peg.1491 | RTY59985.1 | WP_120452023.1 | *flgI* | Flagellar P-ring protein |
| fig\|2497684.3.peg.1492 | RTY59986.1 | WP_120452025.1 | *flgJ* | Flagellar protein [peptidoglycan hydrolase] |
| fig\|2497684.3.peg.1493 | RTY59987.1 | WP_120452027.1 | *flgK* | Flagellar hook-associated protein |
| fig\|2497684.3.peg.1494 | RTY59988.1 | WP_120452029.1 | *flgL* | Flagellar hook-associated protein |
| fig\|2497684.3.peg.1880 | RTY60498.1 | WP_126688973.1 | *aer* | Aerotaxis sensor receptor protein |
| fig\|2497684.3.peg.2058 | RTY54512.1 | WP_120453572.1 | *fliR* | Flagellar biosynthesis protein |
| fig\|2497684.3.peg.2059 | RTY54513.1 | WP_120453574.1 | *fliQ* | Flagellar biosynthesis protein |
| fig\|2497684.3.peg.2060 | RTY54564.1 | WP_126690519.1 | *fliP* | Flagellar biosynthesis protein |
| fig\|2497684.3.peg.2061 | RTY54565.1 | WP_120454200.1 | *fliO* | Flagellar biosynthesis protein |
| fig\|2497684.3.peg.2062 | RTY54514.1 | WP_120453576.1 | *fliN* | Flagellar motor switch protein |
| fig\|2497684.3.peg.2063 | RTY54515.1 | WP_033749825.1 | *fliM* | Flagellar motor switch protein |
| fig\|2497684.3.peg.2064 | RTY54516.1 | WP_120453578.1 | *fliL* | Flagellar basal body-associated protein |
| fig\|2497684.3.peg.2065 | RTY54517.1 | WP_126690500.1 | *fliK* | Flagellar hook-length control protein |
| fig\|2497684.3.peg.2066 | RTY54518.1 | WP_120453581.1 | *fliJ* | Flagellar protein |
| fig\|2497684.3.peg.2067 | RTY54519.1 | WP_120453583.1 | *fliI* | Flagellum-specific ATP synthase |
| fig\|2497684.3.peg.2068 | RTY54520.1 | WP_126690501.1 | *fliH* | Flagellar assembly protein |
| fig\|2497684.3.peg.2069 | RTY54521.1 | WP_120453587.1 | *fliG* | Flagellar motor switch protein |
| fig\|2497684.3.peg.2070 | RTY54522.1 | WP_126690502.1 | *fliF* | Flagellar M-ring protein |
| fig\|2497684.3.peg.2071 | RTY54523.1 | WP_120453591.1 | *fliE* | Flagellar hook-basal body complex protein |
| fig\|2497684.3.peg.2092 | RTY54537.1 | WP_120453751.1 | *fliT* | Flagellar biosynthesis protein |
| fig\|2497684.3.peg.2093 | RTY54538.1 | WP_120453753.1 | *fliS* | Flagellar biosynthesis protein |
| fig\|2497684.3.peg.2094 | RTY54539.1 | WP_120453755.1 | *fliD* | Flagellar cap protein |
| fig\|2497684.3.peg.2096 | RTY54540.1 | WP_120453757.1 | *fliC* | Flagellin |
| fig\|2497684.3.peg.2099 | RTY54542.1 | WP_120453761.1 |  | RNA polymerase sigma factor for flagellar operon |
| fig\|2497684.3.peg.2100 | RTY54543.1 | WP_048785856.1 | *fliZ* | Regulator of sigma S factor |
| fig\|2497684.3.peg.2116 | RTY54557.1 | WP_120453785.1 | *flhD* | Flagellar transcriptional activator |
| fig\|2497684.3.peg.2117 | RTY54558.1 | WP_120453787.1 | *flhC* | Flagellar transcriptional activator |
| fig\|2497684.3.peg.2119 | RTY54559.1 | WP_120453789.1 | *motA* | Flagellar motor rotation protein |
| fig\|2497684.3.peg.2120 | RTY54560.1 | WP_120453791.1 | *motB* | Flagellar motor rotation protein |
| fig\|2497684.3.peg.2121 | RTY54561.1 | WP_126690517.1 | *cheA* | Signal transduction histidine kinase |
| fig\|2497684.3.peg.2122 | RTY54562.1 | WP_120453794.1 | *cheW* | Positive regulator of CheA protein activity |
| fig\|2497684.3.peg.3232 | RTY58614.1 | WP_126689231.1 | *cheV* | Chemotaxis protein (EC 2.7.3.-) |
| fig\|2497684.3.peg.4035 | RTY57577.1 | WP_126689680.1 | *cheY* | Chemotaxis regulator - transmits chemoreceptor signals to flagellar motor components |
| fig\|2497684.3.peg.4052 | RTY57592.1 | WP_120456358.1 | *fliD* | Flagellar cap protein |
| fig\|2497684.3.peg.4427 | RTY57330.1 | WP_120457013.1 | *hdfR* | LysR family transcriptional regulator |
| fig\|2497684.3.peg.4623 | RTY57330.1 |  | *fliC* | Flagellin |
| fig\|2497684.3.peg.660 | RTY55337.1 | WP_120453800.1 | *cheR* | Chemotaxis protein methyltransferase (EC 2.1.1.80) |
| fig\|2497684.3.peg.661 | RTY55338.1 | WP_120453802.1 | *cheB* | Chemotaxis response regulator protein-glutamate methylesterase (EC 3.1.1.61) |
| fig\|2497684.3.peg.662 | RTY55339.1 | WP_120453804.1 | *cheY* | Chemotaxis regulator - transmits chemoreceptor signals to flagellar motor components |
| fig\|2497684.3.peg.663 | RTY55340.1 | WP_120453806.1 | *cheZ* | Chemotaxis response - phosphatase |
| fig\|2497684.3.peg.664 | RTY55341.1 | WP_120453808.1 | *flhB* | Flagellar biosynthesis protein |
| fig\|2497684.3.peg.665 | RTY55342.1 | WP_120453809.1 | *flhA* | Flagellar biosynthesis protein |
| fig\|2497684.3.peg.657 | RTY55334.1 |  | *mcp* | Methyl-accepting chemotaxis protein I (serine chemoreceptor protein) |
| fig\|2497684.3.peg.658 | RTY55335.1 | WP_126690335.1 | *mcp* | Methyl-accepting chemotaxis protein I (serine chemoreceptor protein) |
| fig\|2497684.3.peg.659 | RTY55336.1 | WP_126690336.1 | *tsr* | methyl-accepting chemotaxis protein I, serine sensor receptor |
| fig\|2497684.3.peg.3230 | RTY58613.1 | WP_126689230.1 | *tar* | methyl-accepting chemotaxis protein II, aspartate sensor receptor |
| fig\|2497684.3.peg.4430 | RTY56915.1 | WP_126689873.1 | *trg* | methyl-accepting chemotaxis protein III, ribose and galactose sensor receptor |
| **Type IV pilus/Fimbriae** | |  |  |  |
| fig\|2497684.3.peg.4250 | RTY57169.1 | WP_120457292.1 |  | 3-dehydroquinate synthase (EC 4.2.3.4) |
| fig\|2497684.3.peg.122 | RTY56625.1 | WP_120454291.1 |  | Leader peptidase (Prepilin peptidase) (EC 3.4.23.43) |
| fig\|2497684.3.peg.2511 | RTY59027.1 | WP_126688990.1 |  | Multimodular transpeptidase-transglycosylase (EC 2.4.1.129) (EC 3.4.-.-) |
| fig\|2497684.3.peg.4258 | RTY57175.1 | WP_126689803.1 |  | Multimodular transpeptidase-transglycosylase (EC 2.4.1.129) (EC 3.4.-.-) |
| fig\|2497684.3.peg.21 | RTY56532.1 | WP_126690055.1 | *pilT* | Twitching motility protein |
| fig\|2497684.3.peg.2553 | RTY59066.1 | WP_126689008.1 | *pilB* | Type IV fimbrial assembly, ATPase |
| fig\|2497684.3.peg.2554 | RTY59067.1 | WP_126689009.1 | *pilC* | Type IV fimbrial assembly protein |
| fig\|2497684.3.peg.2552 | RTY59065.1 | WP_120455744.1 | *pilA* | Type IV pilin |
| fig\|2497684.3.peg.4257 | RTY59179.1 | WP_120455544.1 | *pilM* | Type IV pilus biogenesis protein |
| fig\|2497684.3.peg.2674 | RTY57174.1 | WP_126689802.1 | *sfmA* | Fimbriae-like adhesin |
| fig\|2497684.3.peg.597 | RTY55641.1 | WP_120454733.1 |  | Sigma-fimbriae uncharacterized paralogous subunit |
| fig\|2497684.3.peg.598 | RTY55642.1 | WP_120454735.1 |  | Sigma-fimbriae uncharacterized paralogous subunit |
| fig\|2497684.3.peg.599 | RTY55643.1 | WP_120454737.1 |  | Sigma-fimbriae chaperone protein |
| fig\|2497684.3.peg.600 | RTY55644.1 | WP_126690312.1 |  | Sigma-fimbriae usher protein |
| fig\|2497684.3.peg.601 | RTY55645.1 | WP_126690313.1 |  | Sigma-fimbriae tip adhesin |
| fig\|2497684.3.peg.3106 | RTY52558.1 | WP_126690831.1 |  | Fimbriae-like adhesin SfmA |
| **Elongation factor** | |  |  |  |
| fig\|2497684.3.peg.4528 | RTY56999.1 | WP_120453397.1 | *fusA* | Translation elongation factor G |
| fig\|2497684.3.peg.4210 | RTY57130.1 | WP_033752983.1 | *fusA* | Translation elongation factor G |
| fig\|2497684.3.peg.3949 | RTY58010.1 | WP_126689616.1 |  | Translation elongation factor LepA |
| fig\|2497684.3.peg.4209 | RTY52358.1 |  |  | Translation elongation factor Tu |
| fig\|2497684.3.peg.3432 | RTY52358.1 |  |  | Translation elongation factor Tu |
| fig\|2497684.3.peg.3438 | RTY52224.1 | WP_126690840.1 |  | Translation elongation factor Tu |
| fig\|2497684.3.peg.2440 | RTY53508.1 |  | *Tuf* | Translation elongation factor Tu |
| fig\|2497684.3.peg.2368 | RTY53508.1 |  | *Tuf* | Translation elongation factor Tu |
| fig\|2497684.3.peg.2751 | RTY59240.1 | WP_120455416.1 |  | Translation initiation factor 2 |

1. **Antimicrobial resistance (AMR) and multidrug efflux pumps**

| **Class** | **PATRIC ID** | **NCBI ID** |  | **Gene** | **Predicted gene product** |
| --- | --- | --- | --- | --- | --- |
| **Antibiotic resistance** | | |  |  |  |
| Bacitracin resistance | fig\|2497684.3.peg.1890 | RTY60500.1 | WP_120452895.1 | *bcrC* | Undecaprenyl-diphosphatase |
|  | fig\|2497684.3.peg.1299 | RTY59809.1 | WP_120451718.1 | *bcrC* | Undecaprenyl-diphosphatase |
| Beta-lactamases | fig\|2497684.3.peg.2538 | RTY59052.1 | WP_126689002.1 |  | Class_A_beta-lactamase |
|  | fig\|2497684.3.peg.4473 | RTY56951.1 | WP_126689892.1 | *phnP* | Metal-dependent hydrolases of the  beta-lactamase superfamily |
| Colicin resistance | fig\|2497684.3.peg.2636 | RTY59142.1 | WP_120455614.1 | *creA* | Tolerance to colicin E2 |
| Bicyclomycin resistance cluster | fig\|2497684.3.peg.1043 | RTY54846.1 | WP_120457967.1 |  | Transcription_termination_factor_Rho |
|  | fig\|2497684.3.peg.4669 | RTY56771.1 | WP_120453149.1 | *Bcr-1* | Multidrug_resistance_transporter |
| Daptomycin Resistance | fig\|2497684.3.peg.1427 | RTY59926.1 | WP_120451941.1 |  | CDP-diacylglycerol--glycerol-3-phosphate 3-phosphatidyltransferase |
|  | fig\|2497684.3.peg.900 | RTY55057.1 | WP_120454025.1 |  | Cardiolipin_synthase,_bacterial_type_ClsA |
|  | fig\|2497684.3.peg.4298 | RTY57211.1 | WP_126689817.1 |  | Glycerophosphoryl_diester_phosphodiesterase |
| Fusaric acid resistance cluster | fig\|2497684.3.peg.1763 | RTY60230.1 | WP_120452440.1 | *fusE* | Tetrapartite_efflux_system,_membrane_fusion_ component |
|  | fig\|2497684.3.peg.1764 | RTY60231.1 | WP_120452442.1 | *fusD* | Tetrapartite_efflux_system_component |
|  | fig\|2497684.3.peg.1762 | RTY60229.1 | WP_126688818.1 | *fusBC* | Tetrapartite_efflux_system,_inner_membrane_component |
| Resistance to fluoroquinolones | fig\|2497684.3.peg.4696 | RTY56791.1 | WP_120453115.1 | *gyrA* | DNA gyrase subunit A |
|  | fig\|2497684.3.peg.202 | RTY56447.1 | WP_126690138.1 | *gyrB* | DNA gyrase subunit B |
| Polymyxin resistance | fig\|2497684.3.peg.1757 | RTY60224.1 | WP_126688815.1 | *eptA/pmrC* | Lipid_A_phosphoethanolamine_transferase |
|  | fig\|2497684.3.peg.407 | RTY55813.1 | WP_126690226.1 | *eptB* | Kdo2-lipid_A_phosphoethanolamine_transferase |
|  | fig\|2497684.3.peg.4190 | RTY57705.1 | WP_126689760.1 | *eptA/pmrC* | Lipid_A_phosphoethanolamine_transferase |
|  | fig\|2497684.3.peg.4026 | RTY57569.1 | WP_120456318.1 | *eptA/pmrC* | Lipid_A_phosphoethanolamine_transferase |
|  | fig\|2497684.3.peg.969 | RTY55118.1 | WP_126690452.1 |  | UDP-4-amino-4-deoxy-L-arabinose_formyltransferase |
|  | fig\|2497684.3.peg.2762 | RTY59250.1 | WP_120455405.1 | *basR/pmr* | Two-component_transcriptional_regulatory_protein |
|  | fig\|2497684.3.peg.970 | RTY55119.1 | WP_120454133.1 | *arnD* | 4-deoxy-4-formamido-L-arabinose-phosphoundecaprenol_deformylase |
|  | fig\|2497684.3.peg.973 | RTY55122.1 | WP_126690455.1 | *arnF* | Undecaprenyl_phosphate-aminoarabinose_flippase_subunit |
|  | fig\|2497684.3.peg.968 | RTY55117.1 | WP_120454130.1 |  | Undecaprenyl-phosphate_4-deoxy-4-formamido-L-arabinose_transferase |
|  | fig\|2497684.3.peg.2763 | RTY59251.1 | WP_120455403.1 | *basS/pmrB* | Sensor_protein |
|  | fig\|2497684.3.peg.972 | RTY55121.1 | WP_126690454.1 | *arnE* | Undecaprenyl_phosphate-aminoarabinose_flippase_subunit |
|  | fig\|2497684.3.peg.967 | RTY55116.1 | WP_120454128.1 |  | UDP-4-amino-4-deoxy-L-arabinose--oxoglutarate_aminotransferase_(EC_2.6.1.87) |
|  | fig\|2497684.3.peg.971 | RTY55120.1 | WP_126690453.1 |  | Undecaprenyl_phosphate-alpha-4-amino-4-deoxy-L-arabinose_arabinosyl_transferase |
| Triclosan Resistance | fig\|2497684.3.peg.958 | RTY55109.1 | WP_120454114.1 |  | Enoyl-[acyl-carrier-protein]_reductase_[NADH] |
| **Multidrug efflux pumps** | | |  |  |  |
| MFS type EmrKY-TolC and EmrAB-TolC | fig\|2497684.3.peg.2266 | RTY53813.1 | WP_126690597.1 | *emrR* | Multidrug resistance regulator |
|  | fig\|2497684.3.peg.2267 | RTY53814.1 | WP_126690598.1 | *emrA* | Multidrug efflux system EmrAB-OMF, membrane fusion component |
|  | fig\|2497684.3.peg.2268 | RTY53815.1 | WP_126690599.1 | *emrB* | Multidrug efflux system EmrAB-OMF, inner-membrane proton/drug antiporter |
| MFS type in Gram-negative bacteria | fig\|2497684.3.peg.1277 | RTY59791.1 | WP_126688603.1 |  | Membrane fusion component of MSF-type tripartite multidrug efflux system |
|  | fig\|2497684.3.peg.1278 | RTY59792.1 | WP_120451681.1 |  | Inner-membrane proton/drug antiporter (MSF type) of tripartite multidrug efflux system |
|  | fig\|2497684.3.peg.1435 | RTY59933.1 | WP_126688677.1 |  | Inner-membrane proton/drug antiporter (MSF type) of tripartite multidrug efflux system |
|  | fig\|2497684.3.peg.1436 | RTY59934.1 | WP_120451957.1 |  | Membrane fusion component of MSF-type tripartite multidrug efflux system |
|  | fig\|2497684.3.peg.3442 | RTY58137.1 | WP_126689327.1 |  | Membrane fusion component of MSF-type tripartite multidrug efflux system |
|  | fig\|2497684.3.peg.3443 | RTY58138.1 | WP_126689328.1 |  | Inner-membrane proton/drug antiporter (MSF type) of tripartite multidrug efflux system |
|  | fig\|2497684.3.peg.3737 | RTY57818.1 | WP_126689500.1 |  | Inner-membrane proton/drug antiporter (MSF type) of tripartite multidrug efflux system |
|  | fig\|2497684.3.peg.3738 | RTY57819.1 | WP_120458200.1 |  | Membrane fusion component of MSF-type tripartite multidrug efflux system |
| RND type AcrAB-TolC | fig\|2497684.3.peg.1192 | RTY59711.1 | WP_120451527.1 |  | AcrZ membrane protein associated with AcrAB-TolC multidrug efflux pump |
|  | fig\|2497684.3.peg.1281 | RTY59795.1 | WP_120451687.1 |  | DNA-binding protein H-NS |
|  | fig\|2497684.3.peg.3342 | RTY58716.1 | WP_120451312.1 |  | Multidrug efflux system AcrAB-TolC, inner-membrane proton/drug antiporter AcrB (RND type) |
|  | fig\|2497684.3.peg.3343 | RTY58717.1 | WP_120451313.1 |  | Multidrug efflux system AcrAB-TolC, membrane fusion component AcrA |
|  | fig\|2497684.3.peg.3344 | RTY58718.1 | WP_120451314.1 |  | Transcriptional regulator of acrAB operon, AcrR |
|  | fig\|2497684.3.peg.3794 | RTY57870.1 | WP_126689526.1 |  | Aminoglycosides efflux system AcrAD-TolC, inner-membrane proton/drug antiporter AcrD (RND type) |
|  | fig\|2497684.3.peg.4137 | RTY57661.1 | WP_120456486.1 |  | DNA-binding protein H-NS |
|  | fig\|2497684.3.peg.889 | RTY55048.1 | WP_120454006.1 |  | DNA-binding protein H-NS |
| RND type MdtABC-TolC | fig\|2497684.3.peg.4607 | RTY57069.1 | WP_120453268.1 |  | Multidrug efflux system MdtABC-TolC, membrane fusion component MdtA |
|  | fig\|2497684.3.peg.4608 | RTY57070.1 | WP_126689967.1 |  | Multidrug efflux system MdtABC-TolC, inner-membrane proton/drug antiporter MdtB (RND type) |
|  | fig\|2497684.3.peg.4609 | RTY57071.1 | WP_126689968.1 |  | Multidrug efflux system MdtABC-TolC, inner-membrane proton/drug antiporter MdtC (RND type) |
|  | fig\|2497684.3.peg.4610 | RTY57072.1 | WP_126689969.1 |  | Uncharacterized transporter MdtD of major facilitator superfamily (MFS) |
|  | fig\|2497684.3.peg.632 | RTY55673.1 | WP_120454787.1 |  | Outer membrane channel TolC (OpmH) |
|  | fig\|2497684.3.peg.89 | RTY56594.1 | WP_126690085.1 |  | Multidrug efflux system MdtABC-TolC, membrane fusion component MdtA |
|  | fig\|2497684.3.peg.90 | RTY56595.1 | WP_126690086.1 |  | Multidrug efflux system MdtABC-TolC, inner-membrane proton/drug antiporter MdtB (RND type) |
|  | fig\|2497684.3.peg.91 | RTY56596.1 | WP_126690087.1 |  | Multidrug efflux system MdtABC-TolC, inner-membrane proton/drug antiporter MdtC (RND type) |
|  | fig\|2497684.3.peg.92 | RTY56597.1 | WP_126690088.1 |  | Outer membrane factor (OMF) lipoprotein associated wth MdtABC efflux system |
| RND efflux system | fig\|2497684.3.peg.1809 | RTY60273.1 | WP_126688837.1 | *oqxB* | multidrug efflux RND transporter permease subunit |
|  | fig\|2497684.3.peg.1810 | RTY60274.1 | WP_126688838.1 |  | membrane fusion protein |
|  | fig\|2497684.3.peg.86 | RTY56591.1 | WP_126690082.1 |  | efflux RND transporter periplasmic adaptor subunit |
|  | fig\|2497684.3.peg.87 | RTY56592.1 | WP_126690083.1 |  | efflux RND transporter permease subunit |
| Multidrug transporter subunit MdtN | fig\|2497684.3.peg.3022 | RTY52822.1 | WP_126690779.1 | *mdtN* | Inner membrane component MdtN of tripartite multidrug resistance system |

**Iron uptake and transport**

| **PATRIC ID** | **NCBI ID** | **Refseq ID** | **Gene** | **Predicted gene product** |
| --- | --- | --- | --- | --- |
| fig\|2497684.3.peg.1527 | RTY60019.1 | WP_120452079.1 | *efeO* | Ferrous iron transport periplasmic protein |
| fig\|2497684.3.peg.1526 | RTY60018.1 | WP_126688715.1 | *efeU* | Ferrous iron transport permease |
| fig\|2497684.3.peg.1528 | RTY60020.1 | WP_120452081.1 | *efeB* | Ferrous iron transport peroxidase |
| fig\|2497684.3.peg.541 | RTY55591.1 | WP_126690287.1 | *hmuS* | Hemin transport protein |
| fig\|2497684.3.peg.3348 | RTY58721.1 | WP_120451318.1 | *ybaN* | Inner membrane protein |
| fig\|2497684.3.peg.4141 | RTY57664.1 | WP_126689743.1 | *iroN* | Outer Membrane Siderophore Receptor |
| fig\|2497684.3.peg.263 | RTY56023.1 | WP_126690161.1 | *iroE* | Periplasmic esterase |
| fig\|2497684.3.peg.3657 | RTY58316.1 | WP_126689440.1 | *IutA* | Aerobactin siderophore receptor |
| fig\|2497684.3.peg.3659 | RTY58318.1 | WP_120456778.1 | *IucC* | Aerobactin synthase (EC 6.3.2.39), aerobactin biosynthesis protein |
| fig\|2497684.3.peg.2509 | RTY59025.1 | WP_120455812.1 | *fhuC* | Ferric hydroxamate ABC transporter (TC 3.A.1.14.3), ATP-binding protein |
| fig\|2497684.3.peg.2508 | RTY59024.1 | WP_126688988.1 | *fhuD* | Ferric hydroxamate ABC transporter (TC 3.A.1.14.3), periplasmic substrate binding protein |
| fig\|2497684.3.peg.2507 | RTY59023.1 | WP_126688987.1 | *fhuB* | Ferric hydroxamate ABC transporter (TC 3.A.1.14.3), permease component |
| fig\|2497684.3.peg.2510 | RTY59026.1 | WP_126688989.1 | *fhuA* | Ferric hydroxamate outer membrane receptor |
| fig\|2497684.3.peg.3663 | RTY58322.1 | WP_126689444.1 |  | Iron-chelator utilization protein |
| fig\|2497684.3.peg.545 | RTY55595.1 | WP_126690291.1 |  | Iron-chelator utilization protein |
| fig\|2497684.3.peg.3658 | RTY58317.1 | WP_120456780.1 | *iucD* | L-lysine 6-monooxygenase [NADPH] (EC 1.14.13.59), aerobactin biosynthesis protein |
| fig\|2497684.3.peg.3659 | RTY58318.1 | WP_120456778.1 | *iucC* | Aerobactin synthase |
| fig\|2497684.3.peg.3660 | RTY58319.1 | WP_126689441.1 | *iucB* | N6-hydroxylysine O-acetyltransferase (EC 2.3.1.102), aerobactin biosynthesis protein IucB |
| fig\|2497684.3.peg.3661 | RTY58320.1 | WP_126689442.1 | *iucA* | N(2)-citryl-N(6)-acetyl-N(6)-hydroxylysine synthase |
| fig\|2497684.3.peg.3662 | RTY58321.1 | WP_126689443.1 |  | Possible H+-antiporter clustered with aerobactin genes |
| fig\|2497684.3.peg.4004 | RTY57551.1 | WP_126689660.1 |  | Enterobactin esterase |
| fig\|2497684.3.peg.2765 | RTY59253.1 | WP_126689100.1 |  | Enterobactin esterase |
| fig\|2497684.3.peg.2771 | RTY59259.1 | WP_126689105.1 | *entS* | Enterobactin exporter |
| fig\|2497684.3.peg.2768 | RTY59256.1 | WP_126689102.1 | *fepC* | Ferric enterobactin transport ATP-binding protein (TC 3.A.1.14.2) |
| fig\|2497684.3.peg.2770 | RTY59258.1 | WP_126689104.1 | *fepD* | Ferric enterobactin transport system permease protein (TC 3.A.1.14.2) |
| fig\|2497684.3.peg.2769 | RTY59257.1 | WP_126689103.1 | *fepG* | Ferric enterobactin transport system permease protein (TC 3.A.1.14.2) |
| fig\|2497684.3.peg.4003 | RTY57720.1 | WP_126689772.1 | *fepB* | Ferric enterobactin-binding periplasmic protein (TC 3.A.1.14.2) |
| fig\|2497684.3.peg.2772 | RTY59260.1 | WP_126689106.1 | *fepB* | Ferric enterobactin-binding periplasmic protein (TC 3.A.1.14.2) |

**Heavy metal resistance**

| **PATRIC ID** | **NCBI ID** | **Gene** | **Refseq ID** | **Product** |
| --- | --- | --- | --- | --- |
| fig\|2497684.3.peg.1180 | RTY59700.1 | *zitB* | WP_120451506.1 | Zinc transporter |
| fig\|2497684.3.peg.2407 | RTY53475.1 | *zntR* | WP_120458459.1 | Zn(2+)-responsive transcriptional regulator |
| fig\|2497684.3.peg.1090 | RTY54659.1 | *cusR* | WP_084884211.1 | Copper-sensing response regulator |
| fig\|2497684.3.peg.1089 | RTY54658.1 | *cusS* | WP_048785848.1 | Copper sensory histidine kinase |
| fig\|2497684.3.peg.1118 | RTY54679.1 | *merR* | WP_075203154.1 | Cd(II)/Pb(II)-responsive transcriptional regulator |
| fig\|2497684.3.peg.2828 | RTY59312.1 | *cutA* | WP_120455299.1 | Periplasmic divalent cation tolerance protein CutA |
| fig\|2497684.3.peg.671 | RTY55347.1 | *cutC* | WP_120453817.1 | Cytoplasmic copper homeostasis protein CutC |
| fig\|2497684.3.peg.2187 | RTY54290.1 | *cutE* | WP_126690547.1 | Copper homeostasis protein CutE |
| fig\|2497684.3.peg.2188 | RTY54291.1 | *corC* | WP_120451410.1 | Magnesium and cobalt efflux protein CorC |
| fig\|2497684.3.peg.2101 | RTY54544.1 | *dcyD* | WP_126690509.1 | Adaptation to D-Cysteine (D-cysteine desulfhydrase)† |
