# Supplementary material for: First Report of Pathogenic Bacterium Kalamiella piersonii Isolated from Urine of a Kidney Stone Patient: Draft Genome and Evidence for Role in Struvite Crystallization
Source: Pathogens. 2020 Aug 29;9(9):711. doi: 10.3390/pathogens9090711 (PMC7558591; doi:10.3390/pathogens9090711)
Supplement: Supplementary file 1 [file pathogens-09-00711-s001.zip › Table S5.docx]

**S6 Table. Genes involved in urea and amino acid metabolism present in the genome of *Kalamiella piersonii* YU22**

| **PATRIC ID** | **NCBI ID** | **Gene** | **Refseq ID** | **Product** |
| --- | --- | --- | --- | --- |
| **Urea utilization** | |  |  |  |
| fig\|2497684.3.peg.1151 | RTY59672.1 | *pxpA* | WP_126688554.1 | 5-oxoprolinase subunit PxpA |
| fig\|2497684.3.peg.3989 | RTY57537.1 | *urtA* | WP_126689646.1 | Urea_ABC_transporter,_substrate-binding_protein_UrtA |
| fig\|2497684.3.peg.3990 | RTY57538.1 | *urtB* | WP_126689647.1 | Urea_ABC_transporter,_permease_protein_UrtB |
| fig\|2497684.3.peg.3991 | RTY57539.1 | *urtC* | WP_126689648.1 | Urea_ABC_transporter,_permease_protein_UrtC |
| fig\|2497684.3.peg.3992 | RTY57540.1 | *urtD* | WP_126689649.1 | Urea_ABC_transporter,_ATPase_protein_UrtD |
| fig\|2497684.3.peg.3993 | RTY57541.1 | *urtE* | WP_126689650.1 | Urea_ABC_transporter,_ATPase_protein_UrtE |
| fig\|2497684.3.peg.1149 | RTY59670.1 | *pxpB* | WP_120451450.1 | 5-oxoprolinase subunit PxpB |
| fig\|2497684.3.peg.2046 | RTY60462.1 |  | WP_126688951.1 | Urea_carboxylase-related_amino_acid_permease |
| **Urea metabolism:** | |  |  |  |
| fig\|2497684.3.peg.3986 | RTY57534.1 | *atzF* | WP_126689643.1 | Allophanate hydrolase |
| fig\|2497684.3.peg.3987 | RTY57535.1 | *uca* | WP_126689644.1 | Urea carboxylase |
| **Uric acid metabolism** |  |  |  |  |
| fig\|2497684.3.peg.513 | RTY55907.1 | *xanP* | WP_120458133.1 | Uracil-xanthine permease |
| fig\|2497684.3.peg.1869 | RTY60322.1 |  | WP_126689411 | Xanthin dehydrogenase |
| fig\|2497684.3.peg.3131 | RTY58528.1 | *puuE* | WP_126689178.1 | Uricase (allantoinase PuuE) |
| **Ammonia metabolism:** | |  |  |  |
| fig\|2497684.3.peg.3329 | RTY58705.1 | *amtB* | WP_120451301.1 | Ammonium transporter |
| fig\|2497684.3.peg.520 | RTY55912.1 |  | WP_120458138.1 | Type 1 Glutamate : ammonia ligase |
| fig\|2497684.3.peg.4556 | RTY57021.1 |  | WP_126689939.1 | Glutamine synthase family protein |
| **Ammonia-forming enzyme** | |  |  |  |
| fig\|2497684.3.peg.768 | RTY55432.1 | *asnB* | WP_120453980.1 | Asparaginase |
| fig\|2497684.3.peg.297 | RTY57628.1 | *glsA* | WP_126690175.1 | Glutaminase |
| fig\|2497684.3.peg.3582 | RTY58254.1 | *gdh* | WP_120456420.1 | Glutamate dehydrogenase |
| fig\|2497684.3.peg.4095 | RTY57628.1 | *gdh* | WP_126689404.1 | Glutamate dehydrogenase |
