# Supplementary material for: First Report of Pathogenic Bacterium Kalamiella piersonii Isolated from Urine of a Kidney Stone Patient: Draft Genome and Evidence for Role in Struvite Crystallization
Source: Pathogens. 2020 Aug 29;9(9):711. doi: 10.3390/pathogens9090711 (PMC7558591; doi:10.3390/pathogens9090711)
Supplement: Supplementary file 1 [file pathogens-09-00711-s001.zip › Table S6.docx]

**S1- Table:** Details of the 10 taxon used for calculation of OrthoANI values

| **Sl. No.** | **Species name** | **Strain** |
| --- | --- | --- |
| 1 | *Kalamiella piersonii* | YU22 |
| 2 | *Kalamiella piersonii* | IIIF1SW-P2 |
| 3 | *Erwinia pyrifoliae* | DSM 12163 |
| 4 | *Erwinia amylovora* | NBRC 12687 |
| 5 | *Erwinia tasmaniensis* | Et1_99 |
| 6 | *Mixta calida* | DSM 22759 |
| 7 | *Mixta gaviniae* | DSM 22758 |
| 8 | *Pantoea agglomerans* | NBRC 102470 |
| 9 | *Pantoea alhagi* | LTYR-11Z |
| 10 | *Pantoea cypripedii* | LMG 2657 |
