# Supplementary figures and images for: First Report of Pathogenic Bacterium Kalamiella piersonii Isolated from Urine of a Kidney Stone Patient: Draft Genome and Evidence for Role in Struvite Crystallization
Source: Pathogens. 2020 Aug 29;9(9):711. doi: 10.3390/pathogens9090711 (PMC7558591; doi:10.3390/pathogens9090711)

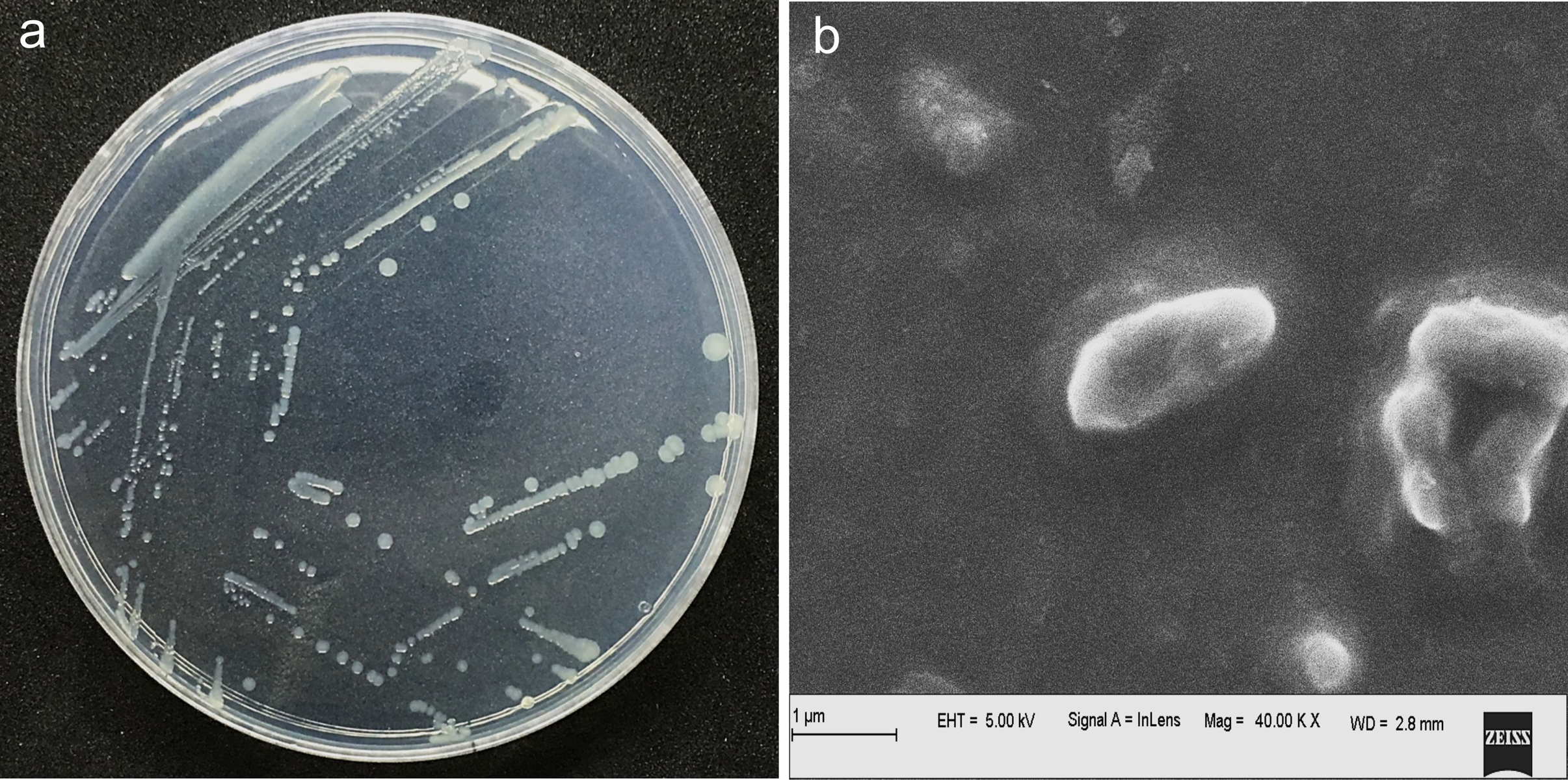

Supplement: Supplementary file 1 [file pathogens-09-00711-s001.zip › Figure S1.tif]

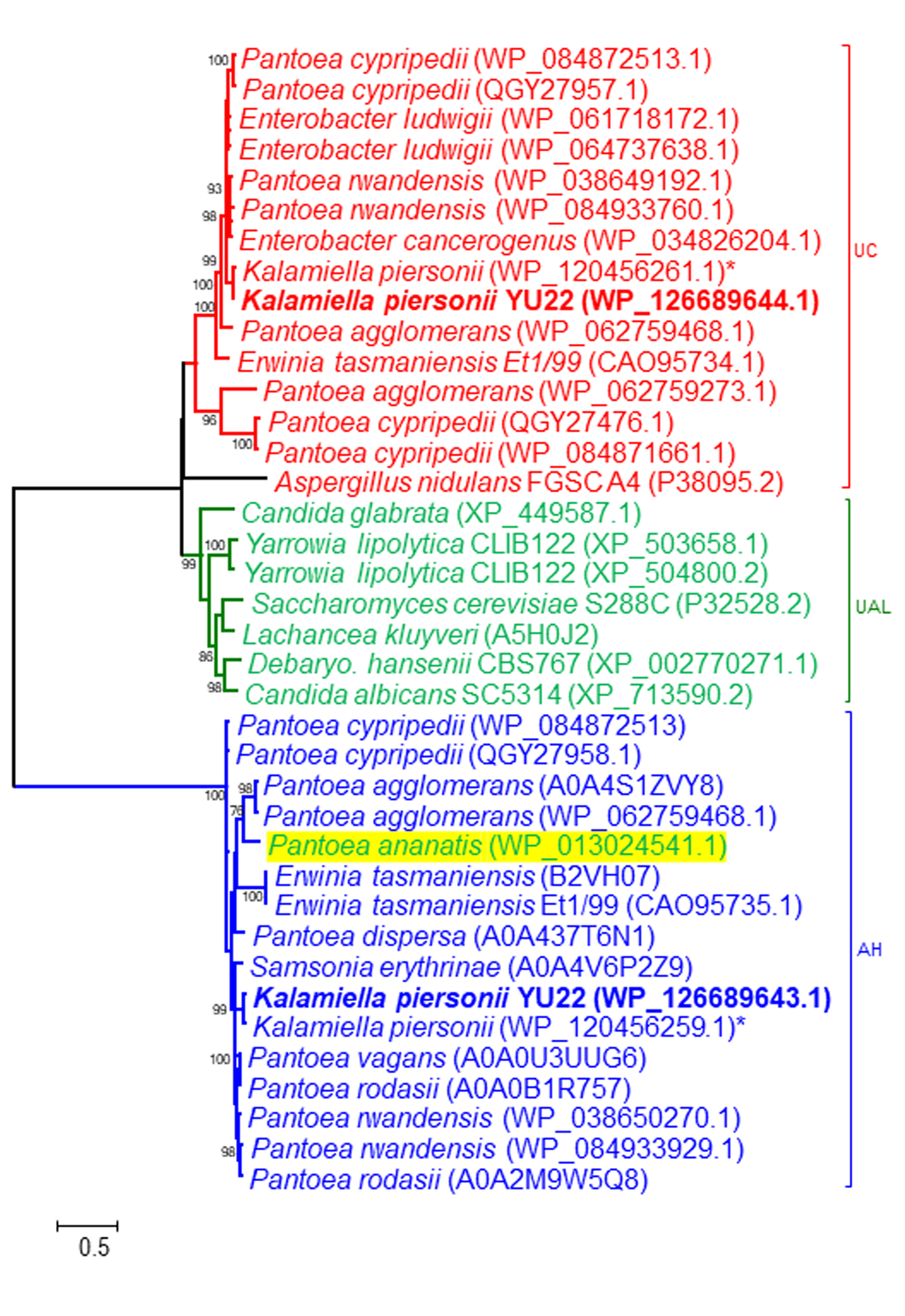

Supplement: Supplementary file 1 [file pathogens-09-00711-s001.zip › Figure S2.tif]
